# Deciphering the Interactions of SARS-CoV-2 Proteins with Human Ion Channels Using Machine-Learning-Based Methods

**DOI:** 10.3390/pathogens11020259

**Published:** 2022-02-17

**Authors:** Nupur S. Munjal, Dikscha Sapra, K. T. Shreya Parthasarathi, Abhishek Goyal, Akhilesh Pandey, Manidipa Banerjee, Jyoti Sharma

**Affiliations:** 1 Institute of Bioinformatics, International Technology Park, Bangalore 560066, India; nupur@ibioinformatics.org (N.S.M.); dikschasapra@gmail.com (D.S.); shreya@ibioinformatics.org (K.T.S.P.); abhishek.goyal.in@gmail.com (A.G.); 2 Center for Molecular Medicine, National Institute of Mental Health and Neurosciences (NIMHANS), Hosur Road, Bangalore 560029, India; pandey.akhilesh@mayo.edu; 3 Department of Laboratory Medicine and Pathology, Mayo Clinic, Rochester, MN 55905, USA; 4 Center for Individualized Medicine, Mayo Clinic, Rochester, MN 55905, USA; 5 Kusuma School of Biological Sciences, Indian Institute of Technology Delhi, Hauz Khas, New Delhi 110016, India; manidipa.banerjee@bioschool.iitd.ac.in; 6 Manipal Academy of Higher Education (MAHE), Udupi 576104, India

**Keywords:** virus and host, protein interaction networks, cellular pathways, antiviral compounds

## Abstract

Severe acute respiratory syndrome coronavirus 2 (SARS-CoV-2) is accountable for the protracted COVID-19 pandemic. Its high transmission rate and pathogenicity led to health emergencies and economic crisis. Recent studies pertaining to the understanding of the molecular pathogenesis of SARS-CoV-2 infection exhibited the indispensable role of ion channels in viral infection inside the host. Moreover, machine learning (ML)-based algorithms are providing a higher accuracy for host-SARS-CoV-2 protein–protein interactions (PPIs). In this study, PPIs of SARS-CoV-2 proteins with human ion channels (HICs) were trained on the PPI-MetaGO algorithm. PPI networks (PPINs) and a signaling pathway map of HICs with SARS-CoV-2 proteins were generated. Additionally, various U.S. food and drug administration (FDA)-approved drugs interacting with the potential HICs were identified. The PPIs were predicted with 82.71% accuracy, 84.09% precision, 84.09% sensitivity, 0.89 AUC-ROC, 65.17% Matthews correlation coefficient score (MCC) and 84.09% F1 score. Several host pathways were found to be altered, including calcium signaling and taste transduction pathway. Potential HICs could serve as an initial set to the experimentalists for further validation. The study also reinforces the drug repurposing approach for the development of host directed antiviral drugs that may provide a better therapeutic management strategy for infection caused by SARS-CoV-2.

## 1. Introduction

The COVID-19 pandemic rapidly spread to more than 175 countries within the first three months of its outbreak [1]. As of October 2021, more than 234 million cases have been reported, including 4.8 million deaths worldwide [2]. The dearth of approved treatments specific for SARS-CoV-2 infection impeded disease containment measures and control of the spread of infection. Viruses with small-sized genomes in particular, depend on the host genomic machinery for many of their essential functions via interacting with membrane proteins [3]. Human ion channels (HICs) are transmembrane proteins that allow the passive flow of ions across cellular membranes owing to their electrochemical gradient. This exchange of ions across the membrane results in electrical currents that contribute to a diverse set of roles, including the generation of membrane potential and cellular activities, such as signal transduction, synaptic release of neurotransmitters, and apoptosis [4]. Numerous viral infections involved in neuronal pathologies, diarrhoea, cardiomyopathies, bronchitis and pain disorders exploit a variety of HICs [3].

Ebola and influenza viruses exploit HICs to enter the host by utilizing Ca^2+^ channels [5,6]. Bunyamwera virus activates host potassium channels during the first six hours of its infection cycle [7]. The use of verapamil, L-type voltage gated Ca^2+^ channel inhibitor, in the treatment of infections caused by filoviruses further implicates the importance of HICs in viral survival [7]. Most likely, in order to survive and replicate inside a host, viruses must exploit the cellular environment, which is highly dependent on the flow of ions into and out of the cell [8]. The entry of SARS-CoV-2 exploits HICs as the conformational changes occurring in viral S protein prior to entering into the endosomes and fusion with the lysosomal membranes is promoted by calcium ions [9,10,11]. This further results in the elevation of cytosolic calcium concentration and aids in viral replication by inhibiting host protein trafficking and the maturation of viral proteins [12,13,14]. Advances in HIC-viral interaction studies provide insights into channelopathies, which may explain some commonly observed virus-induced pathologies [3]. The systematic mapping of PPIs of SARS-CoV-2 and human proteins was studied by Gordon et al. for exploring host dependencies of the SARS-CoV-2 [8]. Additionally, PPIs between SARS-CoV-2 proteins and human proteins implementing ML approaches were studied [15,16,17]. ML-based approaches in predicting interactions allow experimentalists to carry out further analysis with an improved efficacy of PPIs [18].

The current study focuses on understanding the interactome of SARS-CoV-2 proteins with HICs through in silico approaches. PPIs of SARS-CoV-2 proteins with HICs were trained on the PPI-MetaGO algorithm to associate the proteins based on its features. Later, PPI networks (PPINs) of SARS-CoV-2 proteins with HICs were generated. Thereafter, a biological pathway analysis of HICs interacting with SARS-CoV-2 proteins was performed and a pathway map depicting the role of HICs upon interactions with SARS-CoV-2 proteins was generated. Furthermore, FDA-approved drugs interacting with potential HICs were identified. The study may provide an insight to better understand the interactome of SARS-CoV-2 proteins with HICs and may unravel the development of future therapeutic strategies against SARS-CoV-2 infection. The study also underlines the potential significance of repurposing of drugs.

## 2. Results

A total of 181 interactions of HICs with SARS-CoV-2 proteins and 21 interactions among SARS-CoV-2 proteins were obtained from the BioGRID database (release 4.92.192) (Table S1). Thus, 404 interactions consisting of positive dataset (202 interactions) and negative dataset (202 interactions) were used as input for PPI-MetaGO.

### 2.1. Predictions of Protein–Protein Interactions of SARS-CoV-2 Proteins with HICs

SARS-CoV-2 and HIC interactions were predicted with an accuracy of 84.09% and AUC of 0.89 (Table 1). Confusion matrix obtained for the dataset represents 37 true positives, 7 false positives, 7 false negatives, and 30 true negatives (Table 2).

### 2.2. Protein–Protein Interaction Networks (PPINs) of HICs with SARS-CoV-2 Proteins

Of the 328 HICs, 40 were found to interact with 15 SARS-CoV-2 proteins. The functions of 40 HICs interacting with SARS-CoV-2 proteins are listed in Table S2.

#### 2.2.1. Protein-Protein Interaction Maps and Networks

**Figure 1:**
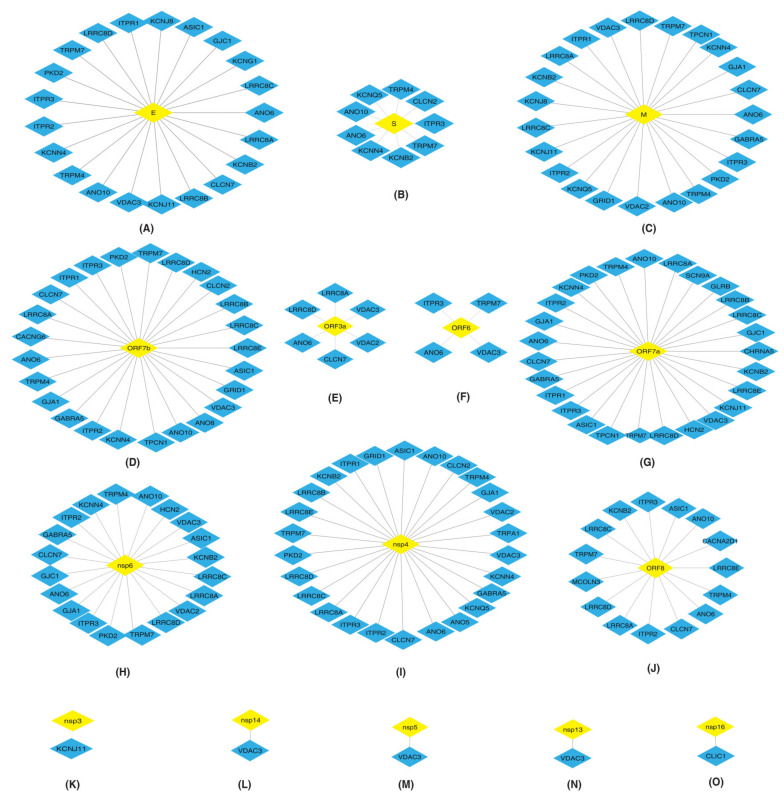
Depiction of protein–protein interactions of SARS-CoV-2 proteins: (**A**) E (**B**) S (**C**) M (**D**) ORF7b (**E**) ORF3a (**F**) ORF6 (**G**) ORF7a (**H**) nsp6 (**I**) nsp4 (**J**) ORF8 (**K**) nsp3 (**L**) nsp14 (**M**) nsp5 (**N**) nsp13 and (**O**) nsp16 with human ion channels (HICs). Yellow color diamond shaped node represents SARS-CoV-2 proteins and HICs are represented as a blue color diamond.

PPI maps of HICs-SARS-CoV-2 proteins are depicted in Figure 1. PPIs of all the SARS-CoV-2 proteins with HICs are shown as a network in Figure S1.

The HICs were found to interact with multiple SARS-CoV-2 proteins.

##### Interactions of Inositol 1,4,5-Trisphosphate Receptors (ITPRs) with SARS-CoV-2 Proteins

ITPRs are the major receptors involved in the release of calcium from the endoplasmic reticulum (ER) [19,20,21,22]. SARS-CoV-2 E, M, ORF7b, ORF7a and nsp4 proteins (Figure 1A,C,D,G,I) interacted with ITPR1, ITPR2 and ITPR3. Nsp6 and ORF8 (Figure 1H,J) interacted with ITPR2 and ITPR3. Additionally, S and ORF6 (Figure 1B,F) interacted with ITPR3.

##### Interactions of Leucine Rich Volume Regulated Anion Channels (LRRCs) with SARS-CoV-2 Proteins

LRRCs are involved in the development and functions of immune cells [23]. E, M, ORF7b, ORF3a, ORF7a, nsp6, nsp4 and ORF8 SARS-CoV-2 proteins (Figure 1A,C–E,G–J) interacted with LRRC8A.

##### Interactions of Voltage Dependent Anion Channels (VDACs) with SARS-CoV-2 Proteins

VDACs control the flow of Ca^2+^ ions from the mitochondrial membrane [24]. M, ORF3a, nsp6 and nsp4 (Figure 1C,D,H,I) interacted with VDAC2 and VDAC3. E, ORF7b, ORF6, ORF7a, nsp14, nsp5 and nsp13 (Figure 1A,D,F,G,L–N) interacted with VDAC3.

##### Interactions of Gap Junctions (GJ) with SARS-CoV-2 Proteins

GJ allow the intercellular flow of Ca2+ ions and enable communication between adjacent cells [25]. M, nsp4, nsp6, ORF7a and ORF7b viral proteins (Figure 1C,D,G–I) interacted with GJA1.

##### Interactions of Anoctamins (ANO) with SARS-CoV-2 Proteins

ANO are calcium-dependent chloride channel proteins. SARS-CoV-2 E, S, M, ORF7b, ORF7a, nsp6, nsp4 and ORF8 (Figure 1A–D,G–J) interacted with ANO6 and ANO10. Additionally, ORF3a and ORF6 (Figure 1E,F) interacted with ANO6.

##### Interactions of Transient Receptor Potential Cation Channels (TRPs) with SARS-CoV-2 Proteins

Proteins belonging to TRP family have a role in signal transductions [26,27,28], indicating their importance in cellular communications. TRPM7 is an ion channel as well as a serine/threonine protein kinase [29]. E, S, M, ORF7b, ORF6, ORF7a, nsp6, nsp4 and ORF8 (Figure 1A–D,F–J) interacted with TRPM7. PKD2 also known as TRPP2, a calcium permeable cation channel, interacted with E, M, ORF7b, ORF7a, nsp6 and nsp4 (Figure 1A,C,D,G–I).

#### 2.2.2. Intra Protein Interactions

**Figure 2:**
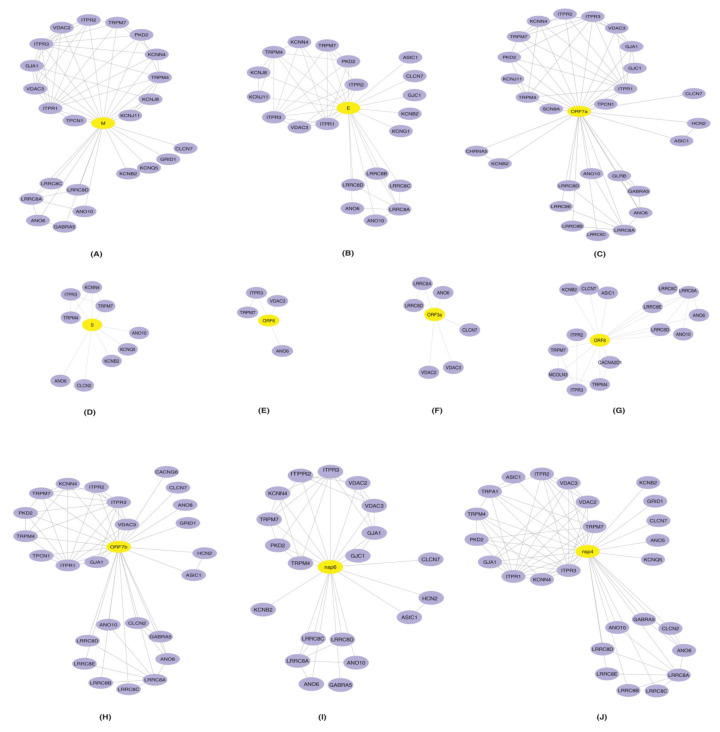
Representation of protein–protein interaction networks of HICs-SARS-CoV-2 proteins: protein–protein interactions of human ion channels (HICs) were generated using STRING database (purple color nodes). Furthermore, HICs interaction networks were overlaid with SARS-CoV-2 proteins (**A**) M, (**B**) E, (**C**) ORF7a (**D**) S, (**E**) ORF6, (**F**) ORF3a, (**G**) ORF8, (**H**) ORF7b, (**I**) nsp6 and (**J**) nsp4. Yellow color node represents SARS-CoV-2 proteins and human ion channels are represented by the purple color node.

The Intra Protein Interactions among the 40 HICs Were Generated as PPINs Using STRING Database (v11) [30] to Identify the HICs that Most Likely Function in an Inter-dependent Manner While Performing a Biological Process. Furthermore, PPINs Were Overlaid with SARS-CoV-2 Proteins and Were Visualized Using Cytoscape (Figure 2).

Several identified HICs were reported in various cellular processes that aid in viral replication and survival. These processes could be the outcome of interactions among the HICs along with the interactions between HICs and SARS-CoV-2 proteins.

##### Role of ITPRs

Viruses exploiting ITPRs were reported to affect the host by increasing metabolic stress and enterotoxicity [31]. Additionally, viral infections promoted depletion of ER Ca^2+^ storage using ITPRs that in turn promoted viral replication [31]. The interaction of SARS-CoV-2 proteins with ITPRs may promote viral replication inside the host cells.

##### Role of LRRCs

LRRCs played important roles in T-cell/ B-cell and lymphocyte function and development [23]. Thus, the identified interactions between LRRC8A and SARS-CoV-2 proteins may play roles in the impairment of T-cell development during the course of infection. These proteins were also reported for their involvement in the maintenance of constant cell volume, efflux of amino acids and import of antibiotic blasticidin-S into the cells [32,33,34,35,36,37].

##### Role of VDACs

VDACs were reported for their involvement in the transportation of metabolites from mitochondria to ER during viral replication and interacted with the structural and non-structural proteins (nsps) of dengue virus [24].

##### Role of GJs

Host cell junctions were reported to be destroyed by viruses during invasion [25]. Another role of GJs reported in viral infection was the amplification of antiviral signaling in neighboring cells. It was studied that STING-dependent recognition was essential for limiting virus replication in the influenza virus [23]. Thus, it could be inferred that gap junctions could act as a port of entry for the virus and further limit the immune reaction against SARS-CoV-2 infection [38]. Most likely, GJA1 may be involved in the cell-to-cell spread of the virus. Additionally, gap junction proteins play a major role in the contraction of the heart [39,40,41]. Thus, they could have a role in the predisposition of the cardiac arrythmias observed during viral infections [42].

##### Role of Other Significant HICs

Other important HICs found were the potassium voltage gated channels that were known to act as modulators of potassium flow into the cell and also had roles in the reactivation of naïve T cells [43,44,45,46,47,48,49,50,51,52,53,54]. The HICs that belonged to voltage-dependent anion channels controlled the flow of Ca^2+^ ions during viral entry [6,24,55]. The chloride voltage gated channels helped to maintain homeostasis and also contributed to acidification, thus maintaining lysosomal pH [55,56,57,58,59]. Anoctamins may be responsible for the entry of viral proteins into the cells [60,61,62,63,64,65,66]. ASIC1, generally involved in learning, pain, sensation, memory and fear [67], was also identified. TPCN1, HCN2, SCN9A, GRID1, CHRNA5 and MCOLN3 were also present. These proteins function as voltage-gated calcium channels across lysosomal membranes, native pacemaker currents in heart, sodium-selective channels that allowed Na^+^ to pass as per the electrochemical gradient, channels at synapses and cation channels for inwardly rectifying activity, respectively [68,69,70,71,72,73,74]. The glycine receptor beta protein found was a member of ligand gated chloride channels [75]. The GABRA5, a component of heteropentameric receptor for gamma-aminobutyric acid (GABA), was involved in GABA-A receptor assembly [76].

### 2.3. Pathway Analysis of 40 HICs Interacting with SARS-CoV-2 Proteins

Pathway analysis of 40 HICs was performed using the STRING database to gather functional insights about the processes regulated by the HICs. Forty-six biological pathways corresponding to the KEGG PATHWAY database were identified. Inflammatory mediator regulation of TRP channels, insulin secretion, renin secretion, pathways involving gap junction, taste transduction, calcium signaling pathway, apelin signaling pathway and GnRH secretion were selected as significant pathways (Table 3).

### 2.4. Signaling Pathway Map of HICs with SARS-CoV-2 Proteins

**Figure 3:**
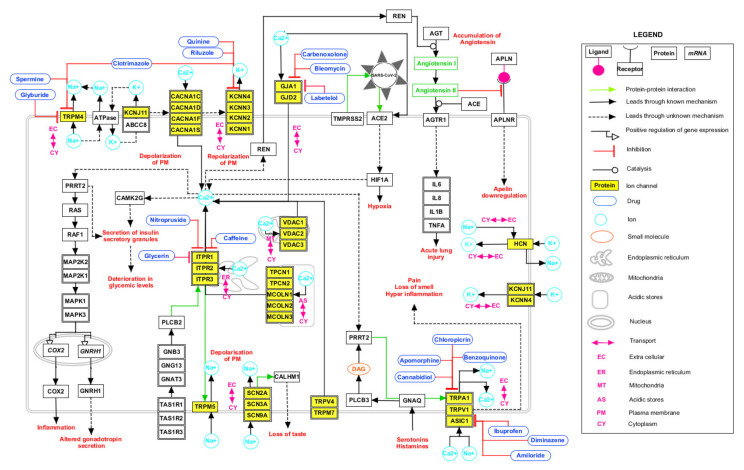
Schematic depiction of possible reactions induced by severe acute respiratory syndrome coronavirus 2 (SARS-CoV-2) via interactions with human ion channels (HICs). The signaling pathway map depicts the biological processes affected upon SARS-CoV-2 infection and the drugs interacting with HICs. The edges representing the relationship between the nodes are provided in the legend.

The advent of omics technologies led to an enormous progress in the generation of signaling pathways [77]. The pathway map of SARS-CoV was generated as a source that could aid in the understanding of viral entry into the cell and the lifecycle of the virus in infected cells [78]. A signaling pathway map for the HICs interacting with SARS-CoV-2 proteins (Figure 3) was generated based on data mining approaches. SARS-CoV-2 entry involves various interactions of the S1-subunit of viral S protein with angiotensin converting enzyme 2 receptors (ACE2) of human cell and transmembrane protease-serine 2 (TMPRSS) receptors that aid in virus–cell membrane fusion. This fusion accompanied by conformational changes of S protein allowed the insertion of fusion peptide into the lipid bilayer and was promoted by Ca^+2^ ions [11]. The interaction of viral proteins with ACE2 may further lead to hypoxic conditions. Renin-angiotensin aldosterone-system, which includes renin as the key regulator in the maintenance of extracellular fluid volume and blood pressure homeostasis, is secreted in the juxta-glomerular cells. This secretion was mainly controlled by free cytosolic Ca^+2^ concentrations. Renin also catalyzed the conversion of AGT to angiotensin I. The release of interleukins may be further stimulated by angiotensin receptors activated upon the conversion of angiotensin I to angiotensin II catalyzed by ACE protein [79]. The orchestration of renin-induced reactions may lead to acute lung injury [79]. Glucose-stimulated insulin secretion is an important mechanism in regulating glucose homeostasis. This process involved the generation of ATP that inhibited ATP-sensitive K^+^ channels and caused voltage-dependent Ca^+2^ influx [80]. The reduction in the number of insulin secretory granules and thus impaired glucose stimulated insulin secretion during the course of infection [81] may be attributed to this mechanism. GJA1 functions in the intracellular transport of Ca^+2^ ions and thus change in the level of Ca^+2^ ions may act as stimuli for communication by GJA1. Various L-type calcium channels (CACNA1C, CACNA1D, CACNA1S and CACNA1F) mediate the influx of intracellular Ca^+2^ ions. Intracellular Ca^+2^ induced the Raf/MEK/ERK pathway that regulated the transcription of *COX2* and led to inflammation reactions [82]. Additionally, the transcription of gonadotropins is regulated through the Raf/MEK/ ERK pathway. The reduced level of testosterone production in COVID-19 patients was associated with altered secretion of gonadotropins [83]. The mobilization of Ca^+2^ from endoplasmic reticulum was regulated by ITPRs and from mitochondria by VDACs. Additionally, calcium was released from Ca^+2^ acidic stores through TPCNs and MCOLNs. Na^+^ and Ca^+2^ ions influx was facilitated by TRP channels and ASIC1. TRP channels may lead to pain, hyperinflammation, and oxidative stress [84]. The influx of K^+^ ions by KCN and HCN channels was stimulated by Na^+^. The PLCB2/ITP3 signaling cascade mediated the transduction of bitter, sweet and umami taste by opening of TRPM5 followed by depolarization of plasma membrane that allowed the influx of Na^+^ ions. Furthermore, Na^+^ influx prevailed due to the SCN membrane channels. SCN channels interacted with CALHM1 and contributed in the mechanism related to taste inception. Thus, the loss of taste occurring in COVID-19 patients may be linked to the deregulation of these channels.

### 2.5. Drugs Interacting with Potential HICs

**Figure 4:**
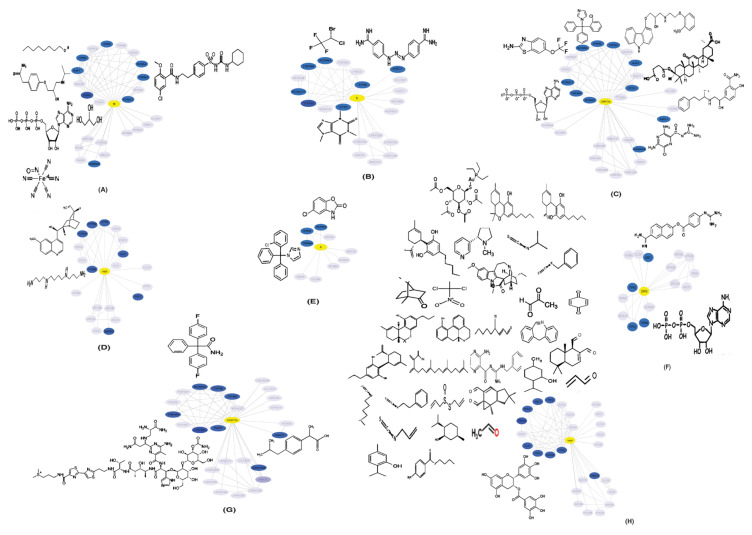
Representation of human ion channels-drug target network: potential human ion channels (HICs) (blue color nodes) interacting with SARS-CoV-2 proteins (**A**) M, (**B**) E, (**C**) ORF7a, (**D**) nsp6, (**E**) S, (**F**) ORF8, (**G**) ORF7b and (**H**) nsp4 (yellow color nodes) and FDA-approved drugs (black) were depicted on HICs-SARS-CoV-2 protein–protein interaction networks.

HICs interacting with viral proteins could be potential drug targets for drug repurposing. Additionally, the traditional drug development method is considerably expensive and time consuming. Drug repurposing is an efficacious process by which effective drugs can be identified. A list of FDA-approved drugs interacting with HICs and SARS-CoV-2 protein is listed in Table S3. Drugs interacting with HICs are depicted on HICs-SARS-CoV-2 PPINs highlighting potential drug targets (Figure 4).

## 3. Discussion

Studies pertaining to the development of novel therapeutic strategies are of utmost importance to combat the heterogeneity in the infection caused by SARS-CoV-2. The current study aimed to understand the interactome of SARS-CoV-2 proteins and HICs through various bioinformatic analyses. It is known that the coronavirus family uses the E protein to induce intracellular membrane remodelling, generating new membrane vesicles that serve as a viral replication site responsible for the depolarisation of membranes [85]. Furthermore, the E protein also helps to bud and release virus particles [85]. Likewise, the M protein along with E protein is responsible for the determination of virion assembly [85]. The nsp3, nsp4 and nsp6, involved in host membrane remodelling and known to act as membrane anchors for replication and transcription complexes, were reported to contain a transmembrane domain [86,87]. This could shed light on the fact that these viral proteins have similar properties as those of HICs and may be involved in host invasion by mimicking the ion channels present in the host. This could be attributed to the presence of a signature sequence in the chlorella virus (PBCV-1) Kcv protein that showed architectural similarity with eukaryotic Kir channels [88]. Hence, it is important to understand the function of viroporins in the manipulation of host-specific processes. However, targeting them can be a challenge due to the resistance polymorphism exhibited by viruses.

Several host pathways were found to be altered due to interactions between SARS-CoV-2 proteins with HICs. Inflammatory mediator regulations of TRP channels pathway include TRP channels (ITPR1, ITPR2 and ITPR3). TRP channels that respond to temperature are known as thermo-TRPs. Among them, TRPA1, TRPM8, and TRPV1-4 are found in the nerve endings and play a major role in pain perception. These proteins are modulated indirectly by inflammatory mediators such as proinflammatory cytokines [89]. The activation of TRPV1 increases the release of several pro-inflammatory molecules, including substance P (sP) and cytokines, such as interleukin-6. Respiratory pathophysiology in SARS-CoV-2 infection may involve TRPV1 receptor sensitization that could result in hyper inflammation of the lungs and associated complications [90]. TRP channels also play a role in the transmission of sensory stimuli of taste [91]. Thus, TRPA1 may increase the sensitivity to evoke pain and several other symptoms associated with SARS-CoV-2 infection [91]. *ACE2* and *TMPRSS2* expression has been reported in the salivary gland cells of the tongue and tonsils. It might allow the virus to fuse its membrane with the host cells as compared to other oral tissues [92]. SARS-CoV-2 may cause changes in the production or quality of saliva, contributing to the symptoms of loss of taste in the oral cavity of infected patients.

Glucose-induced insulin secretion is the main principle of insulin release [80]. Deterioration in glycemic levels including both insulin resistance and impaired insulin secretion were recently reported upon SARS-CoV-2 infection [93,94]. Additionally, a recent study showed that *ACE2* expression increased considerably in human pancreatic beta cells in response to inflammatory cytokines, thus rendering the beta cells more susceptible to SARS-CoV-2 infections [81]. Hence, the roles of HICs, including TRPM4, KCNN4, KCNJ11 and ITPR3, need to be explored further regarding this mechanism.

Gap junctions contain the intercellular channels that allow a direct communication between the cellular compartments. These channels permit the transfer of ions, amino acids, secondary messengers and other metabolites between adjacent cells. Changes in the intracellular Ca^2+^ levels act as stimuli to the gap junctions. ITPRs (ITPR1, ITPR2, ITPR3) play a crucial role in maintaining the intracellular Ca^2+^as they act on the ER for the regulation of cytoplasmic calcium concentration [95].

The renin-angiotensin-aldosterone system (RAAS) is an essential system for electrolyte homeostasis and blood pressure management through the ACE2 axis. The deregulation of RAAS homeostasis results in the development of distress in lungs, induced apoptosis, vasoconstriction, increased oxidative stress and edema [79]. ACE2 acts as a port of entry for SARS-CoV-2 and its expression decreases as the infection progresses [96]. The reduction in expression levels of ACE2 could be correlated to the increase in Ca^2+^-concentration-dependent metalloproteinase domain-containing protein (ADAM10). Moreover, the increase in Ca^2+^ concentration could further be attributed to viral proteins interacting with ITPR3 [97]. Additionally, the decrease in ACE2 level led to the accumulation of angiotensin II, which further activated the angiotensin II type 1 receptor (AT1R) axis, thus worsening the disease outcome. Additionally, apelin signaling could also be suggested to be involved in disease progression. Apelin peptides are endogenous ligands of G protein coupled receptors APJ. Apelin played a number of roles in the mammalian system by protecting cardiac health and calcium modulation [98]. Experimental studies exhibited that apelin administration had anti-inflammatory effects [97].

HICs interacting with viral proteins could be potential drug targets for drug repurposing. The identification of drugs that could be targeted against potential HICs was performed. However, drugs targeted against HICs can be toxic in some case [99]. Therefore, the identified drugs should be tested for the antiviral activity accordingly.

## 4. Material and Methods

**Figure 5:**
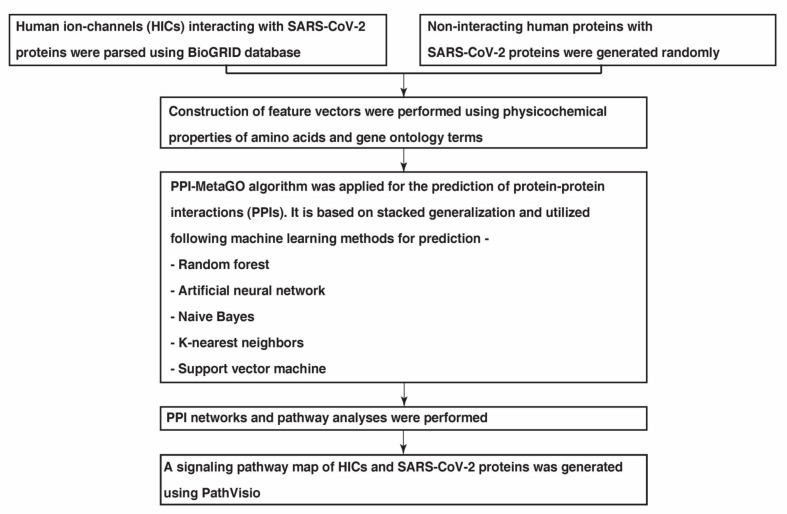
A schematic overview of the several analyses carried out to study the interactome networks of SARS-CoV-2 proteins with human ion channels.

An overview of the methodology followed to study the PPIs between SARS-CoV-2 and HICs is described in Figure 5.

### 4.1. Data Collection

One of the most crucial steps while building any model based on a ML algorithm is the extraction and enhancement of a good dataset. Primarily, a list of 28 unique SARS-CoV-2 proteins were downloaded from the RefSeq database and 328 HICs were retrieved from HGNC database [100]. Interactions between HICs and SARS-CoV-2 proteins were parsed using the BioGRID database (release 4.92.192). These interactions were considered as the positive dataset that were used as input for PPI-MetaGO. The binding affinity of each of the interactions predicted using In SiLico protein AffiNity predictor (ISLAND) [101] was included to scale the affinity between the interactors. There are no ‘gold standard’ negative datasets available. Therefore, for PPI prediction, usually protein pairs are chosen randomly from the set of protein pairs that are not known to interact, and treated as a negative dataset [102]. Hence, in this study for the negative set, first the complement graph of the positive interactions was made and random interactions were taken to generate the negative set. Furthermore, the protein sequences were parsed using RefSeq database and gene ontology terms for SARS-CoV-2 proteins were downloaded from Gene Ontology knowledgebase [103,104].

### 4.2. Feature Extraction and Stacked Generalisation Method for Model Generation

The PPI-MetaGO algorithm [105] was applied for the extraction of features of the protein pairs. It is an ensemble supervised meta learner algorithm for PPI prediction. It employs a hybrid feature representation combining the protein sequence properties, gene ontology information, and interaction network topology. The feature vectors consisting of physicochemical properties of proteins were extracted using protein sequences. The semantic similarities were extracted using the provided GO terms [105]. The dataset was split into a training and testing set in 80:20 ratios. Thereafter, protein sequences and the GO terms were provided as input to the PPI-MetaGO program for the calculation of features and model building. Customized Python scripts were used for the generation of input for PPI-MetaGO. PPI-MetaGO uses a stacked generalization method that allows combining multiple ML algorithms to maximize accuracy. The usage of a single ML-based method may lead to the overfitting or underfitting of data even when the parameters are optimized maximally. Bagging and boosting, which allow multiple ML-based algorithms, only permit the combination of algorithms of the same type and focus on reducing the variance from multiple classifiers [105]. Stacked generalization uses a meta-ML model that allows the combination of different algorithms and aims to reduce the bias of the base generalizers [106]. ML-based methods utilized by PPI-MetaGO include random forest (RF), artificial neural network (ANN), Naïve Bayes (NB), K-nearest neighbors (kNN) and support vector machine (SVM). RF consists of a set of decision trees/base regression trees that form an aggregated regression estimate. kNN classifier classifies unlabeled observations by assigning them to a class with the most similar labeled observation through calculation of distances between two datapoints. NB is a probability-based classifier that classifies a datapoint based on prior probability and likelihood calculations. ANN is a biologically inspired computational network that generates a pattern from the input dataset and provides an output based on those patterns. SVM is supervised learning algorithm that can simultaneously minimize the classification error and maximize the geometric margin between two classes. PPI-MetaGO uses a two-level stacked classifier approach where the bottom layer uses RF, kNN, NB and ANN. The top layer uses SVM with RBF kernel. The predictions from the bottom layer are passed as meta data for the top layer which makes the final prediction.

### 4.3. Evaluation of Predicted PPIs

To evaluate the performance of PPI-MetaGO, a 10-fold cross-validation was performed [105]. Additionally, the PPIs were evaluated using the following performance measures:

$$\mathrm{Accuracy}=\frac{\mathrm{TP}+\mathrm{TN}}{\mathrm{TP}+\mathrm{TN}+\mathrm{FP}+\mathrm{FN}}$$

$$\mathrm{Precision}=\frac{\mathrm{TP}}{\mathrm{TP}+\mathrm{FP}}$$

$$F-\mathrm{score}=\frac{2\;\times \;\mathrm{TPR}\;\times \;\mathrm{Precision}}{\mathrm{TPR}+\mathrm{Precision}}$$

$$\mathrm{MCC}=\frac{\mathrm{TP}\;\times \;\mathrm{TN}-\mathrm{FP}\;\times \;\mathrm{FN}}{\surd \left(\mathrm{TP}+\mathrm{FP})(\mathrm{TP}+\mathrm{FN})(\mathrm{TN}+\mathrm{FP})(\mathrm{TN}+\mathrm{FN}\right)}$$

$$\mathrm{Sensitivity}=\frac{\mathrm{TP}}{\mathrm{TP}+\mathrm{FN}}$$

$$\mathrm{False}\;\mathrm{Positive}\;\mathrm{Rate}=\frac{\mathrm{FP}}{\mathrm{FP}+\mathrm{TN}}$$

where TPR, TP, TN, FP, and FN represent true positive rate, true positive, true negative, false positive, and false negative, respectively. In addition, PPI-MetaGO also calculates area under the curve (AUC). AUC is the probability that a random positive sample will have a higher score than a random negative sample.

### 4.4. Generation of PPI Maps

The PPIs among HICs-SARS-CoV-2 proteins and the PPI maps were visualized using Cytoscape-3.8 [107]. Additionally, PPINs of HICs interacting with SARS-CoV-2 proteins were generated using the STRING database (v11) [30]. Thereafter, the visualization of PPINs of HICs-SARS-CoV-2 proteins was performed using Cytoscape-3.8.

### 4.5. Pathway Analysis of HICs Interacting with SARS-CoV-2 Proteins

The KEGG PATHWAY analysis of HICs interacting with SARS-CoV-2 proteins was performed using the STRING database (v11). Significant pathways were selected based on statistical measures including strength and false discovery rate (FDR) score provided by *p*-values for further analysis.

### 4.6. Generation of Signaling Pathway Map of HICs with SARS-CoV-2 Proteins

An extensive literature search was performed using PubMed to annotate reactions such as protein–protein interactions, translocations, activations and inhibitions that describe HICs in association with SARS-CoV-2 proteins [78,108]. Several query terms were used including, “SARS-CoV-2” AND “ion channels”, “SARS-CoV-2” AND “human ion channels”, “SARS-CoV-2” AND “pathway” OR “signaling”. The articles were screened for information pertaining to biological pathways involving HICs interacting with SARS-CoV-2 proteins. The processes occurring in SARS-CoV-2 infected patients as compared to healthy individuals were included. Thereafter, a signaling pathway map of HICs interacting with SARS-CoV-2 was generated in Graphical Pathway Markup Language (GPML) format using PathVisio (version 3.3.0) [109], an opensource pathway drawing software. In the map, nodes describe the entity pool (proteins and genes) and edges represent the relationship between nodes.

### 4.7. Identification of Drugs Interacting with Potential HICs

FDA-approved drugs interacting with HICs were parsed using the Drug–Gene Interaction database (DGIdb) [110,111]. Furthermore, drugs interacting with HICs were depicted on HICs-SARS-CoV-2 PPINs to highlight potential drug targets.

## 5. Conclusions

Several computational approaches, including ML-based algorithms, were applied to study the interactome of SARS-CoV-2 proteins with HICs. Biological insights of HICs interacting with SARS-CoV-2 proteins were gained using pathway analysis. TRPM4 and KCNN4 were found to a play role in insulin secretion. TRPA1 was found as an important molecule in heat, pain, and taste sensitivity inside the host. GJA1 has a crucial role in pathways involving gap junctions and ASIC1 was found to be a part of inflammatory mediator regulation of TRP channels. ITPR1 was found to be involved in six predicted pathways, including inflammatory mediator regulation of TRP channels, gap junction, renin secretion and apelin signaling pathways. Moreover, FDA-approved drugs interacting with potential HICs were identified that can be repurposed. Most likely, our analyses showed promising results that further require experimental validation. HICs could be further explored as a potential class of targets for the better management of the infection caused by SARS-CoV-2.

## Figures and Tables

**Table 1:** Overall performance of PPI-MetaGO.

Accuracy	Precision	F1 Score	AUC-ROC	MCC	Sensitivity	False Positive Rate
82.71	84.09	84.09	0.89	65.17	84.09	18.91

**Table 2:** Confusion matrix obtained from PPI-MetaGO.

	True Positive	True Negative
**Predicted Positive**	37	7
**Predicted Negative**	7	30

**Table 3:** List of KEGG pathways and potential target HICs.

KEGG Pathway	Potential Target Proteins	Strength	False Discovery Rate
Inflammatory mediator regulation of TRP channels	ASIC1, TRPA1, ITPR1, ITPR2, ITPR3	1.46	0.0000328
Insulin secretion	TRPM4, KCNN4, KCNJ11, ITPR3	1.4	0.00022
Renin secretion	ITPR1, ITPR3, ITPR2	1.4	0.0012
Gap junction	GJA1, ITPR1, ITPR3, ITPR2	1.39	0.00022
Taste transduction	GABRA5, ITPR3, SCN9A	1.29	0.0017
Calcium signaling pathway	ITPR1, MCOLN3, VDAC2, ITPR3, ITPR2, VDAC3, TPCN1	1.2	0.0000511
Apelin signaling pathway	ITPR1, ITPR3, ITPR2	1.08	0.004
GnRH secretion	HCN2, ITPR1, ITPR2, ITPR3, KCNN4, KCNJ11	1.67	0.00000024

## Data Availability

Publicly available datasets were analyzed in this study. The data can be accessible using following resources: RefSeq database (https://www.ncbi.nlm.nih.gov/refseq/, accessed on 18 March 2021), BioGRID database (https://thebiogrid.org/, accessed on 3 March 2021) and Gene Ontology knowledgebase (http://geneontology.org/, accessed on 18 March 2021). Customized Python scripts used for PPI data analysis and the HICs-SARS-CoV-2 pathway reaction data are available in GPML format through GitHub repository (https://github.com/js-iob/HICs-SARS-CoV-2_PPIs/blob/main/HICs_SARS_CoV2_Signaling_Pathway_map.gpml and https://github.com/js-iob/HICs-SARS-CoV-2_PPIs/blob/main/HICs_SARS_CoV2_data_files.rar, accessed on 20 October 2021).

## Supplementary Materials

The following supporting information can be downloaded at: https://www.mdpi.com/article/10.3390/pathogens11020259/s1, Figure S1: Schematic depiction of interactions of total SARS-CoV-2 proteins with human ion channels; Table S1: A list of protein-protein interactions of SARS-CoV-2—human ion channels and SARS-CoV-2—SARS-CoV-2 proteins from BioGRID database (release 4.92.192) with the experimental evidences; Table S2: Function of human ion channels interacting with SARS-CoV-2 proteins; Table S3: A list of drugs interacting with HICs.

## References

[1] Huang C., Wang Y., Li X., Ren L., Zhao J., Hu Y., Zhang L., Fan G., Xu J., Gu X. (2020). Clinical features of patients infected with 2019 novel coronavirus in Wuhan, China. Lancet.

[2] Coronavirus Resource Center. https://coronavirus.jhu.edu/.

[3] Charlton F.W., Pearson H.M., Hover S., Lippiat J.D., Fontana J., Barr J.N., Mankouri J. (2020). Ion Channels as Therapeutic Targets for Viral Infections: Further Discoveries and Future Perspectives. Viruses.

[4] Kim J.B. (2014). Channelopathies. Korean J. Pediatr..

[5] Simmons J.A., D’Souza R.S., Ruas M., Galione A., Casanova J.E., White J.M. (2015). Ebolavirus Glycoprotein Directs Fusion through NPC1+ Endolysosomes. J. Virol..

[6] Fujioka Y., Nishide S., Ose T., Suzuki T., Kato I., Fukuhara H., Fujioka M., Horiuchi K., Satoh A.O., Nepal P. (2018). A Sialylated Voltage-Dependent Ca(2+) Channel Binds Hemagglutinin and Mediates Influenza A Virus Entry into Mammalian Cells. Cell Host Microbe.

[7] Hover S., Foster B., Barr J.N., Mankouri J. (2017). Viral dependence on cellular ion channels—An emerging anti-viral target?. J. Gen. Virol..

[8] Gordon D.E., Jang G.M., Bouhaddou M., Xu J., Obernier K., White K.M., O’Meara M.J., Rezelj V.V., Guo J.Z., Swaney D.L. (2020). A SARS-CoV-2 protein interaction map reveals targets for drug repurposing. Nature.

[9] Li F. (2016). Structure, Function, and Evolution of Coronavirus Spike Proteins. Annu. Rev. Virol..

[10] Perlman S., Netland J. (2009). Coronaviruses post-SARS: Update on replication and pathogenesis. Nat. Rev. Microbiol..

[11] Navarese E.P., Musci R.L., Frediani L., Gurbel P.A., Kubica J. (2020). Ion channel inhibition against COVID-19: A novel target for clinical investigation. Cardiol. J..

[12] Jayaseelan V.P., Paramasivam A. (2020). Repurposing calcium channel blockers as antiviral drugs. J. Cell Commun. Signal..

[13] Wang H., Yang P., Liu K., Guo F., Zhang Y., Zhang G., Jiang C. (2008). SARS coronavirus entry into host cells through a novel clathrin- and caveolae-independent endocytic pathway. Cell Res..

[14] Glebov O.O. (2020). Understanding SARS-CoV-2 endocytosis for COVID-19 drug repurposing. FEBS J..

[15] Dey L., Chakraborty S., Mukhopadhyay A. (2020). Machine learning techniques for sequence-based prediction of viral-host interactions between SARS-CoV-2 and human proteins. Biomed. J..

[16] Nourani E., Khunjush F., Durmus S. (2015). Computational approaches for prediction of pathogen-host protein-protein interactions. Front. Microbiol..

[17] Mei S., Zhang K. (2019). In silico unravelling pathogen-host signaling cross-talks via pathogen mimicry and human protein-protein interaction networks. Comput. Struct. Biotechnol. J..

[18] Sarkar D., Saha S. (2019). Machine-learning techniques for the prediction of protein-protein interactions. J. Biosci..

[19] Atakpa P., Thillaiappan N.B., Mataragka S., Prole D.L., Taylor C.W. (2018). IP3 Receptors Preferentially Associate with ER-Lysosome Contact Sites and Selectively Deliver Ca(2+) to Lysosomes. Cell Rep..

[20] Wiel C., Lallet-Daher H., Gitenay D., Gras B., Le Calve B., Augert A., Ferrand M., Prevarskaya N., Simonnet H., Vindrieux D. (2014). Endoplasmic reticulum calcium release through ITPR2 channels leads to mitochondrial calcium accumulation and senescence. Nat. Commun..

[21] Vervloessem T., Yule D.I., Bultynck G., Parys J.B. (2015). The type 2 inositol 1,4,5-trisphosphate receptor, emerging functions for an intriguing Ca(2)(+)-release channel. Biochim. Biophys. Acta.

[22] Kuchay S., Saeed M., Giorgi C., Li J., Hoffmann H.H., Pinton P., Rice C.M., Pagano M. (2018). NS5A Promotes Constitutive Degradation of IP3R3 to Counteract Apoptosis Induced by Hepatitis C Virus. Cell Rep..

[23] Platt C.D., Chou J., Houlihan P., Badran Y.R., Kumar L., Bainter W., Poliani P.L., Perez C.J., Dent S.Y.R., Clapham D.E. (2017). Leucine-rich repeat containing 8A (LRRC8A)-dependent volume-regulated anion channel activity is dispensable for T-cell development and function. J. Allergy Clin. Immunol..

[24] Jitobaom K., Tongluan N., Smith D.R. (2016). Involvement of voltage-dependent anion channel (VDAC) in dengue infection. Sci. Rep..

[25] Dong D., Xie W., Liu M. (2020). Alteration of cell junctions during viral infection. Thorac. Cancer.

[26] Zhao J., Lin King J.V., Paulsen C.E., Cheng Y., Julius D. (2020). Irritant-evoked activation and calcium modulation of the TRPA1 receptor. Nature.

[27] Xian W., Wang H., Moretti A., Laugwitz K.L., Flockerzi V., Lipp P. (2020). Domain zipping and unzipping modulates TRPM4’s properties in human cardiac conduction disease. FASEB J..

[28] Jha R.M., Desai S.M., Zusman B.E., Koleck T.A., Puccio A.M., Okonkwo D.O., Park S.Y., Shutter L.A., Kochanek P.M., Conley Y.P. (2019). Downstream TRPM4 Polymorphisms Are Associated with Intracranial Hypertension and Statistically Interact with ABCC8 Polymorphisms in a Prospective Cohort of Severe Traumatic Brain Injury. J. Neurotrauma.

[29] Lee C.T., Ng H.Y., Kuo W.H., Tain Y.L., Leung F.F., Lee Y.T. (2020). The role of TRPM7 in vascular calcification: Comparison between phosphate and uremic toxin. Life Sci..

[30] Szklarczyk D., Gable A.L., Lyon D., Junge A., Wyder S., Huerta-Cepas J., Simonovic M., Doncheva N.T., Morris J.H., Bork P. (2019). STRING v11: Protein-protein association networks with increased coverage, supporting functional discovery in genome-wide experimental datasets. Nucleic Acids Res..

[31] Clark K.B., Eisenstein E.M. (2013). Targeting host store-operated Ca(2+) release to attenuate viral infections. Curr. Top. Med. Chem..

[32] Chen L., Konig B., Liu T., Pervaiz S., Razzaque Y.S., Stauber T. (2019). More than just a pressure relief valve: Physiological roles of volume-regulated LRRC8 anion channels. Biol. Chem..

[33] Lu J., Xu F., Zhang J. (2019). Inhibition of angiotensin II-induced cerebrovascular smooth muscle cell proliferation by LRRC8A downregulation through suppressing PI3K/AKT activation. Hum. Cell.

[34] Voss F.K., Ullrich F., Munch J., Lazarow K., Lutter D., Mah N., Andrade-Navarro M.A., von Kries J.P., Stauber T., Jentsch T.J. (2014). Identification of LRRC8 heteromers as an essential component of the volume-regulated anion channel VRAC. Science.

[35] Lutter D., Ullrich F., Lueck J.C., Kempa S., Jentsch T.J. (2017). Selective transport of neurotransmitters and modulators by distinct volume-regulated LRRC8 anion channels. J. Cell Sci..

[36] Lee C.C., Freinkman E., Sabatini D.M., Ploegh H.L. (2014). The protein synthesis inhibitor blasticidin s enters mammalian cells via leucine-rich repeat-containing protein 8D. J. Biol. Chem..

[37] Schober A.L., Wilson C.S., Mongin A.A. (2017). Molecular composition and heterogeneity of the LRRC8-containing swelling-activated osmolyte channels in primary rat astrocytes. J. Physiol..

[38] Ahn J., Barber G.N. (2019). STING signaling and host defense against microbial infection. Exp. Mol. Med..

[39] Martins-Marques T., Catarino S., Goncalves A., Miranda-Silva D., Goncalves L., Antunes P., Coutinho G., Leite Moreira A., Falcao Pires I., Girao H. (2020). EHD1 Modulates Cx43 Gap Junction Remodeling Associated With Cardiac Diseases. Circ. Res..

[40] Brink P.R., Valiunas V., White T.W. (2020). Lens Connexin Channels Show Differential Permeability to Signaling Molecules. Int. J. Mol. Sci..

[41] Ye W.G., Yue B., Aoyama H., Kim N.K., Cameron J.A., Chen H., Bai D. (2017). Junctional delay, frequency, and direction-dependent uncoupling of human heterotypic Cx45/Cx43 gap junction channels. J. Mol. Cell Cardiol..

[42] Guzik T.J., Mohiddin S.A., Dimarco A., Patel V., Savvatis K., Marelli-Berg F.M., Madhur M.S., Tomaszewski M., Maffia P., D’Acquisto F. (2020). COVID-19 and the cardiovascular system: Implications for risk assessment, diagnosis, and treatment options. Cardiovasc. Res..

[43] Fu J., Dai X., Plummer G., Suzuki K., Bautista A., Githaka J.M., Senior L., Jensen M., Greitzer-Antes D., Manning Fox J.E. (2017). Kv2.1 Clustering Contributes to Insulin Exocytosis and Rescues Human beta-Cell Dysfunction. Diabetes.

[44] Yan L., Figueroa D.J., Austin C.P., Liu Y., Bugianesi R.M., Slaughter R.S., Kaczorowski G.J., Kohler M.G. (2004). Expression of voltage-gated potassium channels in human and rhesus pancreatic islets. Diabetes.

[45] Mederos Y.S.M., Rinne S., Skrobek L., Renigunta V., Schlichthorl G., Derst C., Gudermann T., Daut J., Preisig-Muller R. (2009). Mutation of histidine 105 in the T1 domain of the potassium channel Kv2.1 disrupts heteromerization with Kv6.3 and Kv6. J. Biol. Chem..

[46] Du Q., Jovanovic S., Tulic L., Sljivancanin D., Jack D.W., Zizic V., Abdul K.S., Tulic I., Jovanovic A. (2013). KATP channels are up-regulated with increasing age in human myometrium. Mech. Ageing Dev..

[47] Delaney J.T., Muhammad R., Blair M.A., Kor K., Fish F.A., Roden D.M., Darbar D. (2012). A KCNJ8 mutation associated with early repolarization and atrial fibrillation. Europace.

[48] Babenko A.P., Gonzalez G., Aguilar-Bryan L., Bryan J. (1998). Reconstituted human cardiac KATP channels: Functional identity with the native channels from the sarcolemma of human ventricular cells. Circ. Res..

[49] Tammaro P., Ashcroft F.M. (2007). A mutation in the ATP-binding site of the Kir6.2 subunit of the KATP channel alters coupling with the SUR2A subunit. J. Physiol..

[50] Cooper P.E., McClenaghan C., Chen X., Stary-Weinzinger A., Nichols C.G. (2017). Conserved functional consequences of disease-associated mutations in the slide helix of Kir6.1 and Kir6.2 subunits of the ATP-sensitive potassium channel. J. Biol. Chem..

[51] Srivastava S., Li Z., Ko K., Choudhury P., Albaqumi M., Johnson A.K., Yan Y., Backer J.M., Unutmaz D., Coetzee W.A. (2006). Histidine phosphorylation of the potassium channel KCa3.1 by nucleoside diphosphate kinase B is required for activation of KCa3.1 and CD4 T cells. Mol. Cell.

[52] Srivastava S., Zhdanova O., Di L., Li Z., Albaqumi M., Wulff H., Skolnik E.Y. (2008). Protein histidine phosphatase 1 negatively regulates CD4 T cells by inhibiting the K+ channel KCa3. Proc. Natl. Acad. Sci. USA.

[53] Maekawa M., Terasaka S., Mochizuki Y., Kawai K., Ikeda Y., Araki N., Skolnik E.Y., Taguchi T., Arai H. (2014). Sequential breakdown of 3-phosphorylated phosphoinositides is essential for the completion of macropinocytosis. Proc. Natl. Acad. Sci. USA.

[54] Zhang Y., Chu X., Liu L., Zhang N., Guo H., Yang F., Liu Z., Dong Y., Bao Y., Zhang X. (2016). Tannic acid activates the Kv7.4 and Kv7.3/7.5 K(+) channels expressed in HEK293 cells and reduces tension in the rat mesenteric arteries. J. Pharm. Pharmacol..

[55] Muller M., Slivinski N., Todd E., Khalid H., Li R., Karwatka M., Merits A., Mankouri J., Tuplin A. (2019). Chikungunya virus requires cellular chloride channels for efficient genome replication. PLoS Negl. Trop. Dis..

[56] Hansen T.H., Yan Y., Ahlberg G., Vad O.B., Refsgaard L., Dos Santos J.L., Mutsaers N., Svendsen J.H., Olesen M.S., Bentzen B.H. (2020). A Novel Loss-of-Function Variant in the Chloride Ion Channel Gene Clcn2 Associates with Atrial Fibrillation. Sci. Rep..

[57] Leisle L., Ludwig C.F., Wagner F.A., Jentsch T.J., Stauber T. (2011). ClC-7 is a slowly voltage-gated 2Cl(-)/1H(+)-exchanger and requires Ostm1 for transport activity. EMBO J..

[58] Bourdin B., Shakeri B., Tetreault M.P., Sauve R., Lesage S., Parent L. (2015). Functional characterization of CaValpha2delta mutations associated with sudden cardiac death. J. Biol. Chem..

[59] Yang L., Katchman A., Morrow J.P., Doshi D., Marx S.O. (2011). Cardiac L-type calcium channel (Cav1.2) associates with gamma subunits. FASEB J..

[60] Oh U., Jung J. (2016). Cellular functions of TMEM16/anoctamin. Pflugers Arch..

[61] Lin H., Jun I., Woo J.H., Lee M.G., Kim S.J., Nam J.H. (2019). Temperature-dependent increase in the calcium sensitivity and acceleration of activation of ANO6 chloride channel variants. Sci. Rep..

[62] Veit M., Koyro K.I., Ahrens B., Bleibaum F., Munz M., Rovekamp H., Andra J., Schreiber R., Kunzelmann K., Sommer A. (2018). Anoctamin-6 regulates ADAM sheddase function. Biochim. Biophys. Acta Mol. Cell Res..

[63] Lin H., Roh J., Woo J.H., Kim S.J., Nam J.H. (2018). TMEM16F/ANO6, a Ca(2+)-activated anion channel, is negatively regulated by the actin cytoskeleton and intracellular MgATP. Biochem. Biophys. Res. Commun..

[64] Jha A., Chung W.Y., Vachel L., Maleth J., Lake S., Zhang G., Ahuja M., Muallem S. (2019). Anoctamin 8 tethers endoplasmic reticulum and plasma membrane for assembly of Ca(2+) signaling complexes at the ER/PM compartment. EMBO J..

[65] Bushell S.R., Pike A.C.W., Falzone M.E., Rorsman N.J.G., Ta C.M., Corey R.A., Newport T.D., Christianson J.C., Scofano L.F., Shintre C.A. (2019). The structural basis of lipid scrambling and inactivation in the endoplasmic reticulum scramblase TMEM16K. Nat. Commun.

[66] Ishihara K., Suzuki J., Nagata S. (2016). Role of Ca(2+) in the Stability and Function of TMEM16F and 16K. Biochemistry.

[67] Waldmann R., Champigny G., Bassilana F., Heurteaux C., Lazdunski M. (1997). A proton-gated cation channel involved in acid-sensing. Nature.

[68] Pena-Oyarzun D., Batista-Gonzalez A., Kretschmar C., Burgos P., Lavandero S., Morselli E., Criollo A. (2020). New emerging roles of Polycystin-2 in the regulation of autophagy. Int. Rev. Cell Mol. Biol..

[69] Brailoiu E., Churamani D., Cai X., Schrlau M.G., Brailoiu G.C., Gao X., Hooper R., Boulware M.J., Dun N.J., Marchant J.S. (2009). Essential requirement for two-pore channel 1 in NAADP-mediated calcium signaling. J. Cell Biol..

[70] Ludwig A., Zong X., Stieber J., Hullin R., Hofmann F., Biel M. (1999). Two pacemaker channels from human heart with profoundly different activation kinetics. EMBO J..

[71] Ahuja S., Mukund S., Deng L., Khakh K., Chang E., Ho H., Shriver S., Young C., Lin S., Johnson J.P. (2015). Structural basis of Nav1.7 inhibition by an isoform-selective small-molecule antagonist. Science.

[72] Huttlin E.L., Bruckner R.J., Paulo J.A., Cannon J.R., Ting L., Baltier K., Colby G., Gebreab F., Gygi M.P., Parzen H. (2017). Architecture of the human interactome defines protein communities and disease networks. Nature.

[73] Coverstone E.D., Bach R.G., Chen L., Bierut L.J., Li A.Y., Lenzini P.A., O’Neill H.C., Spertus J.A., Sucharov C.C., Stitzel J.A. (2018). A novel genetic marker of decreased inflammation and improved survival after acute myocardial infarction. Basic Res. Cardiol..

[74] Martina J.A., Lelouvier B., Puertollano R. (2009). The calcium channel mucolipin-3 is a novel regulator of trafficking along the endosomal pathway. Traffic.

[75] Handford C.A., Lynch J.W., Baker E., Webb G.C., Ford J.H., Sutherland G.R., Schofield P.R. (1996). The human glycine receptor beta subunit: Primary structure, functional characterisation and chromosomal localisation of the human and murine genes. Brain Res. Mol. Brain Res..

[76] Butler K.M., Moody O.A., Schuler E., Coryell J., Alexander J.J., Jenkins A., Escayg A. (2018). De novo variants in GABRA2 and GABRA5 alter receptor function and contribute to early-onset epilepsy. Brain.

[77] Sharma J., Balakrishnan L., Kaushik S., Kashyap M.K. (2020). Editorial: Multi-Omics Approaches to Study Signaling Pathways. Front. Bioeng. Biotechnol..

[78] Parthasarathi K.T.S., Munjal N.S., Dey G., Kumar A., Pandey A., Balakrishnan L., Sharma J. (2021). A pathway map of signaling events triggered upon SARS-CoV infection. J. Cell Commun. Signal..

[79] Gao Y.L., Du Y., Zhang C., Cheng C., Yang H.Y., Jin Y.F., Duan G.C., Chen S.Y. (2020). Role of Renin-Angiotensin System in Acute Lung Injury Caused by Viral Infection. Infect. Drug Resist..

[80] Komatsu M., Takei M., Ishii H., Sato Y. (2013). Glucose-stimulated insulin secretion: A newer perspective. J. Diabetes Investig..

[81] Muller J.A., Gross R., Conzelmann C., Kruger J., Merle U., Steinhart J., Weil T., Koepke L., Bozzo C.P., Read C. (2021). SARS-CoV-2 infects and replicates in cells of the human endocrine and exocrine pancreas. Nat. Metab..

[82] Ghasemnejad-Berenji M., Pashapour S. (2020). SARS-CoV-2 and the Possible Role of Raf/MEK/ERK Pathway in Viral Survival: Is This a Potential Therapeutic Strategy for COVID-19?. Pharmacology.

[83] Selvaraj K., Ravichandran S., Krishnan S., Radhakrishnan R.K., Manickam N., Kandasamy M. (2021). Testicular Atrophy and Hypothalamic Pathology in COVID-19: Possibility of the Incidence of Male Infertility and HPG Axis Abnormalities. Reprod. Sci..

[84] Jaffal S.M., Abbas M.A. (2021). TRP channels in COVID-19 disease: Potential targets for prevention and treatment. Chem. Biol. Interact..

[85] Arya R., Kumari S., Pandey B., Mistry H., Bihani S.C., Das A., Prashar V., Gupta G.D., Panicker L., Kumar M. (2020). Structural insights into SARS-CoV-2 proteins. J. Mol. Biol..

[86] Snijder E.J., Decroly E., Ziebuhr J. (2016). The Nonstructural Proteins Directing Coronavirus RNA Synthesis and Processing. Adv. Virus Res..

[87] Angelini M.M., Akhlaghpour M., Neuman B.W., Buchmeier M.J. (2013). Severe acute respiratory syndrome coronavirus nonstructural proteins 3, 4, and 6 induce double-membrane vesicles. mBio.

[88] Wang K., Xie S., Sun B. (2010). Viral proteins function as ion channels. Biochim. Biophys. Acta.

[89] Parenti A., De Logu F., Geppetti P., Benemei S. (2016). What is the evidence for the role of TRP channels in inflammatory and immune cells?. Br. J. Pharmacol..

[90] Groneberg D.A., Niimi A., Dinh Q.T., Cosio B., Hew M., Fischer A., Chung K.F. (2004). Increased expression of transient receptor potential vanilloid-1 in airway nerves of chronic cough. Am. J. Respir. Crit. Care Med..

[91] Talavera K. (2015). TRP Channels as Targets for Modulation of Taste Transduction.

[92] Huang N., Perez P., Kato T., Mikami Y., Okuda K., Gilmore R.C., Conde C.D., Gasmi B., Stein S., Beach M. (2021). SARS-CoV-2 infection of the oral cavity and saliva. Nat. Med..

[93] Wu L., Girgis C.M., Cheung N.W. (2020). COVID-19 and diabetes: Insulin requirements parallel illness severity in critically unwell patients. Clin. Endocrinol..

[94] Gianchandani R., Esfandiari N.H., Ang L., Iyengar J., Knotts S., Choksi P., Pop-Busui R. (2020). Managing Hyperglycemia in the COVID-19 Inflammatory Storm. Diabetes.

[95] Ivanova H., Vervliet T., Missiaen L., Parys J.B., De Smedt H., Bultynck G. (2014). Inositol 1,4,5-trisphosphate receptor-isoform diversity in cell death and survival. Biochim. Biophys. Acta.

[96] Saeedi Saravi S.S., Beer J.H. (2020). Apelin-potential therapy for COVID-19?. J. Mol. Cell Cardiol..

[97] Scialo F., Daniele A., Amato F., Pastore L., Matera M.G., Cazzola M., Castaldo G., Bianco A. (2020). ACE2: The Major Cell Entry Receptor for SARS-CoV. Lung.

[98] Wang C., Liu N., Luan R., Li Y., Wang D., Zou W., Xing Y., Tao L., Cao F., Wang H. (2013). Apelin protects sarcoplasmic reticulum function and cardiac performance in ischaemia-reperfusion by attenuating oxidation of sarcoplasmic reticulum Ca2+-ATPase and ryanodine receptor. Cardiovasc. Res..

[99] Feng J.Y., Murakami E., Zorca S.M., Johnson A.A., Johnson K.A., Schinazi R.F., Furman P.A., Anderson K.S. (2004). Relationship between antiviral activity and host toxicity: Comparison of the incorporation efficiencies of 2’,3’-dideoxy-5-fluoro-3’-thiacytidine-triphosphate analogs by human immunodeficiency virus type 1 reverse transcriptase and human mitochondrial DNA polymerase. Antimicrob. Agents Chemother..

[100] Bruford E.A., Lush M.J., Wright M.W., Sneddon T.P., Povey S., Birney E. (2008). The HGNC Database in 2008: A resource for the human genome. Nucleic Acids Res..

[101] Abbasi W.A., Yaseen A., Hassan F.U., Andleeb S., Minhas F. (2020). ISLAND: In-silico proteins binding affinity prediction using sequence information. BioData Min..

[102] Ben-Hur A., Noble W.S. (2006). Choosing negative examples for the prediction of protein-protein interactions. BMC Bioinform..

[103] Ashburner M., Ball C.A., Blake J.A., Botstein D., Butler H., Cherry J.M., Davis A.P., Dolinski K., Dwight S.S., Eppig J.T. (2000). Gene ontology: Tool for the unification of biology. The Gene Ontology Consortium. Nat. Genet..

[104] Seth Carbon E.D., Douglass E., Good B.M., Unni D.R., Harris N.L., Mungall C.J., Basu S., Chisholm R.L., Dodson R.J., Hartline E. (2021). The Gene Ontology resource: Enriching a GOld mine. Nucleic Acids Res..

[105] Chen K.H., Wang T.F., Hu Y.J. (2019). Protein-protein interaction prediction using a hybrid feature representation and a stacked generalization scheme. BMC Bioinform..

[106] Ma Z., Wang P., Gao Z., Wang R., Khalighi K. (2018). Ensemble of machine learning algorithms using the stacked generalization approach to estimate the warfarin dose. PLoS ONE.

[107] Shannon P., Markiel A., Ozier O., Baliga N.S., Wang J.T., Ramage D., Amin N., Schwikowski B., Ideker T. (2003). Cytoscape: A software environment for integrated models of biomolecular interaction networks. Genome Res..

[108] Zhong J., Sharma J., Raju R., Palapetta S.M., Prasad T.S., Huang T.C., Yoda A., Tyner J.W., van Bodegom D., Weinstock D.M. (2014). TSLP signaling pathway map: A platform for analysis of TSLP-mediated signaling. Database.

[109] Kutmon M., van Iersel M.P., Bohler A., Kelder T., Nunes N., Pico A.R., Evelo C.T. (2015). PathVisio 3: An extendable pathway analysis toolbox. PLoS Comput. Biol..

[110] Griffith M., Griffith O.L., Coffman A.C., Weible J.V., McMichael J.F., Spies N.C., Koval J., Das I., Callaway M.B., Eldred J.M. (2013). DGIdb: Mining the druggable genome. Nat. Methods.

[111] Freshour S.L., Kiwala S., Cotto K.C., Coffman A.C., McMichael J.F., Song J.J., Griffith M., Griffith O.L., Wagner A.H. (2021). Integration of the Drug-Gene Interaction Database (DGIdb 4.0) with open crowdsource efforts. Nucleic Acids Res..

