# Supplementary material for: Deciphering the Interactions of SARS-CoV-2 Proteins with Human Ion Channels Using Machine-Learning-Based Methods
Source: Pathogens. 2022 Feb 17;11(2):259. doi: 10.3390/pathogens11020259 (PMC8874499; doi:10.3390/pathogens11020259)
Supplement: Supplementary file 1 [file pathogens-11-00259-s001.zip › pathogens-1557033-supplementary.pdf]

Deciphering the Interactions of SARS-CoV-2 Proteins with Human Ion Channels Using Machine Learning-Based Method

Nupur S. Munjal [1], Diksha Sapra [1], K. T. Shreya Parthasarathi [1], Abhishek Goyal [1], Akhilesh Pandey [2,3,4], Manidipa Banerjee [5] and Jyoti Sharma [1,6,*]

[1] Institute of Bioinformatics, International Technology Park, Bangalore 560066, India; nupur@ibioinformatics.org (N.S.M.); dikschasapra@gmail.com (D.S.); shreya@ibioinformatics.org (K.T.S.P.); abhishek.goyal.in@gmail.com (A.G.)
[2] Center for Molecular Medicine, National Institute of Mental Health and Neurosciences (NIMHANS), Hosur Road, Bangalore 560029, India; pandey.akhilesh@mayo.edu
[3] Department of Laboratory Medicine and Pathology, Mayo Clinic, Rochester, MN 55905, USA
[4] Center for Individualized Medicine, Mayo Clinic, Rochester, MN 55905, USA
[5] Kusuma School of Biological Sciences, Indian Institute of Technology Delhi, Hauz Khas, New Delhi 110016, India; manidipa.banerjee@bioschool.iitd.ac.in
[6] Manipal Academy of Higher Education (MAHE), Manipal, Karnataka 576104, India
* Correspondence: jyoti@ibioinformatics.org

Input Dataset for PPI-MetaGO

193 PPIs of SARS-CoV-2 proteins with HICs were parsed from BioGRID database (release 4.92.192). The interactions of ORF14 and ORF3b with HICs were removed from the input. The dataset included 202 interactions; 181 from SARS-CoV-2 - HICs interactions and 21 from SARS-CoV-2 - SARS-CoV-2 interactions.

Table S1: A list of protein-protein interactions of SARS-CoV-2 - human ion channels and SARS-CoV-2 - SARS-CoV-2 proteins from BioGRID database (release 4.92.192) with the experimental evidences.

Official Symbol Interactor A	Official Symbol Interactor B	Experimental System	Author	Publication Source	Predicted binding affinity in kcal/mol (ISLAND)
E	ANO6	Proximity Label-MS	Samavarchi-Tehrani P (2020)	DOI:10.1101/2020.09.03.282103	-10.727
E	ITPR2	Proximity Label-MS	Samavarchi-Tehrani P (2020)	DOI:10.1101/2020.09.03.282103	-10.568
E	ITPR3	Proximity	Samavarchi-Tehrani P	DOI:10.1101/20	-10.469

		Label-MS	(2020)	20.09.03.282103	
E	KCNN4	Proximity Label-MS	Samavarchi-Tehrani P (2020)	DOI:10.1101/2020.09.03.282103	-10.879
E	PKD2	Proximity Label-MS	Samavarchi-Tehrani P (2020)	DOI:10.1101/2020.09.03.282103	-10.617
E	TRPM4	Proximity Label-MS	Samavarchi-Tehrani P (2020)	DOI:10.1101/2020.09.03.282103	-11.092
E	TRPM7	Proximity Label-MS	Samavarchi-Tehrani P (2020)	DOI:10.1101/2020.09.03.282103	-10.87
E	ANO10	Proximity Label-MS	Samavarchi-Tehrani P (2020)	DOI:10.1101/2020.09.03.282103	-10.444
E	LRRC8D	Proximity Label-MS	Samavarchi-Tehrani P (2020)	DOI:10.1101/2020.09.03.282103	-11.13
E	VDAC3	Proximity Label-MS	Samavarchi-Tehrani P (2020)	DOI:10.1101/2020.09.03.282103	-11.223
E	ITPR1	Proximity Label-MS	Laurent E (2020)	DOI:10.1101/2020.08.28.272955	-10.247
E	KCNJ11	Proximity Label-MS	Laurent E (2020)	DOI:10.1101/2020.08.28.272955	-11.158
E	KCNJ8	Proximity Label-MS	Laurent E (2020)	DOI:10.1101/2020.08.28.272955	-10.835
E	LRRC8B	Proximity Label-MS	Laurent E (2020)	DOI:10.1101/2020.08.28.272955	-10.87
E	ASIC1	Proximity Label-MS	St-Germain JR (2020)	DOI:10.1101/2020.08.28.269175	-11.201
E	CLCN7	Proximity	St-Germain JR	DOI:10.1101/20	-10.519

		Label-MS	(2020)	20.08.28.269175	
E	GJC1	Proximity Label-MS	St-Germain JR (2020)	DOI:10.1101/2020.08.28.269175	-10.366
E	KCNB2	Proximity Label-MS	St-Germain JR (2020)	DOI:10.1101/2020.08.28.269175	-10.87
E	KCNG1	Proximity Label-MS	St-Germain JR (2020)	DOI:10.1101/2020.08.28.269175	-10.344
E	LRRC8A	Proximity Label-MS	St-Germain JR (2020)	DOI:10.1101/2020.08.28.269175	-10.568
E	LRRC8C	Proximity Label-MS	St-Germain JR (2020)	DOI:10.1101/2020.08.28.269175	-10.835
M	ANO6	Affinity Capture-MS	Gordon DE (2020)	PUBMED:32353859	-10.471
M	CLCN7	Proximity Label-MS	Samavarchi-Tehrani P (2020)	DOI:10.1101/2020.09.03.282103	-10.221
M	GABRA5	Proximity Label-MS	Samavarchi-Tehrani P (2020)	DOI:10.1101/2020.09.03.282103	-10.738
M	GJA1	Proximity Label-MS	Samavarchi-Tehrani P (2020)	DOI:10.1101/2020.09.03.282103	-10.21
M	ITPR3	Proximity Label-MS	Samavarchi-Tehrani P (2020)	DOI:10.1101/2020.09.03.282103	-10.379
M	KCNN4	Proximity Label-MS	Samavarchi-Tehrani P (2020)	DOI:10.1101/2020.09.03.282103	-10.422
M	PKD2	Proximity Label-MS	Samavarchi-Tehrani P (2020)	DOI:10.1101/2020.09.03.282103	-10.368
M	TPCN1	Proximity Label-MS	Samavarchi-Tehrani P	DOI:10.1101/2020.09.03.282103	-10.421

			(2020)		
M	TRPM4	Proximity Label-MS	Samavarchi-Tehrani P (2020)	DOI:10.1101/2020.09.03.282103	-10.896
M	ANO10	Proximity Label-MS	Samavarchi-Tehrani P (2020)	DOI:10.1101/2020.09.03.282103	-10.091
M	VDAC2	Proximity Label-MS	Samavarchi-Tehrani P (2020)	DOI:10.1101/2020.09.03.282103	-10.962
M	VDAC3	Proximity Label-MS	Samavarchi-Tehrani P (2020)	DOI:10.1101/2020.09.03.282103	-10.676
M	GRID1	Proximity Label-MS	Laurent E (2020)	DOI:10.1101/2020.08.28.272955	-10.838
M	ITPR1	Proximity Label-MS	Laurent E (2020)	DOI:10.1101/2020.08.28.272955	-10.162
M	KCNQ5	Proximity Label-MS	Laurent E (2020)	DOI:10.1101/2020.08.28.272955	-10.87
M	LRRC8A	Proximity Label-MS	Laurent E (2020)	DOI:10.1101/2020.08.28.272955	-10.283
M	ITPR2	Proximity Label-MS	St-Germain JR (2020)	DOI:10.1101/2020.08.28.269175	-10.48
M	KCNB2	Proximity Label-MS	St-Germain JR (2020)	DOI:10.1101/2020.08.28.269175	-10.87
M	KCNJ11	Proximity Label-MS	St-Germain JR (2020)	DOI:10.1101/2020.08.28.269175	-10.676
M	KCNJ8	Proximity Label-MS	St-Germain JR (2020)	DOI:10.1101/2020.08.28.269175	-10.348
M	LRRC8C	Proximity Label-MS	St-Germain JR (2020)	DOI:10.1101/2020.08.28.269175	-10.565
M	LRRC8D	Proximity	St-Germain JR	DOI:10.1101/20	-10.881

		Label-MS	(2020)	20.08.28.269175	
M	TRPM7	Proximity Label-MS	St-Germain JR (2020)	DOI:10.1101/2020.08.28.269175	-10.87
S	ANO6	Proximity Label-MS	Samavarchi-Tehrani P (2020)	DOI:10.1101/2020.09.03.282103	-11.72
S	KCNN4	Proximity Label-MS	Samavarchi-Tehrani P (2020)	DOI:10.1101/2020.09.03.282103	-11.982
S	TRPM4	Proximity Label-MS	Samavarchi-Tehrani P (2020)	DOI:10.1101/2020.09.03.282103	-11.878
S	CLCN2	Proximity Label-MS	Laurent E (2020)	DOI:10.1101/2020.08.28.272955	-11.823
S	KCNQ5	Proximity Label-MS	Laurent E (2020)	DOI:10.1101/2020.08.28.272955	-10.87
S	ANO10	Proximity Label-MS	St-Germain JR (2020)	DOI:10.1101/2020.08.28.269175	-11.677
S	ITPR3	Proximity Label-MS	St-Germain JR (2020)	DOI:10.1101/2020.08.28.269175	-10.988
S	KCNB2	Proximity Label-MS	St-Germain JR (2020)	DOI:10.1101/2020.08.28.269175	-10.87
S	TRPM7	Proximity Label-MS	St-Germain JR (2020)	DOI:10.1101/2020.08.28.269175	-10.87
ORF3a	ANO6	Affinity Capture-MS	Stukalov A (2020)	DOI:10.1101/2020.06.17.156455	-10.717
ORF3a	LRRC8D	Affinity Capture-MS	Stukalov A (2020)	DOI:10.1101/2020.06.17.156455	-11.124
ORF3a	CLCN7	Affinity Capture-MS	Stukalov A (2020)	DOI:10.1101/2020.06.17.156455	-10.505
ORF3a	LRRC8A	Affinity Capture-MS	Stukalov A (2020)	DOI:10.1101/2020.06.17.156455	-10.555

ORF3a	VDAC2	Proximity Label-MS	Samavarchi-Tehrani P (2020)	DOI:10.1101/2020.09.03.282103	-11.454
ORF3a	VDAC3	Proximity Label-MS	Samavarchi-Tehrani P (2020)	DOI:10.1101/2020.09.03.282103	-11.219
ORF6	ANO6	Proximity Label-MS	Samavarchi-Tehrani P (2020)	DOI:10.1101/2020.09.03.282103	-10.402
ORF6	ITPR3	Proximity Label-MS	Samavarchi-Tehrani P (2020)	DOI:10.1101/2020.09.03.282103	-10.355
ORF6	VDAC3	Proximity Label-MS	Samavarchi-Tehrani P (2020)	DOI:10.1101/2020.09.03.282103	-10.509
ORF6	TRPM7	Proximity Label-MS	St-Germain JR (2020)	DOI:10.1101/2020.08.28.269175	-10.87
ORF7a	CLCN7	Proximity Label-MS	Samavarchi-Tehrani P (2020)	DOI:10.1101/2020.09.03.282103	-9.934
ORF7a	ANO6	Proximity Label-MS	Samavarchi-Tehrani P (2020)	DOI:10.1101/2020.09.03.282103	-10.218
ORF7a	GABRA5	Proximity Label-MS	Samavarchi-Tehrani P (2020)	DOI:10.1101/2020.09.03.282103	-10.26
ORF7a	GJA1	Proximity Label-MS	Samavarchi-Tehrani P (2020)	DOI:10.1101/2020.09.03.282103	-9.747
ORF7a	ITPR1	Proximity Label-MS	Samavarchi-Tehrani P (2020)	DOI:10.1101/2020.09.03.282103	-10.078
ORF7a	ITPR2	Proximity Label-MS	Samavarchi-Tehrani P	DOI:10.1101/2020.09.03.282103	-10.392

			(2020)		
ORF7a	ITPR3	Proximity Label-MS	Samavarchi-Tehrani P (2020)	DOI:10.1101/2020.09.03.282103	-10.289
ORF7a	KCNN4	Proximity Label-MS	Samavarchi-Tehrani P (2020)	DOI:10.1101/2020.09.03.282103	-9.969
ORF7a	ASIC1	Proximity Label-MS	Samavarchi-Tehrani P (2020)	DOI:10.1101/2020.09.03.282103	-10.518
ORF7a	PKD2	Proximity Label-MS	Samavarchi-Tehrani P (2020)	DOI:10.1101/2020.09.03.282103	-10.124
ORF7a	TPCN1	Proximity Label-MS	Samavarchi-Tehrani P (2020)	DOI:10.1101/2020.09.03.282103	-10.14
ORF7a	TRPM4	Proximity Label-MS	Samavarchi-Tehrani P (2020)	DOI:10.1101/2020.09.03.282103	-10.695
ORF7a	ANO10	Proximity Label-MS	Samavarchi-Tehrani P (2020)	DOI:10.1101/2020.09.03.282103	-9.756
ORF7a	LRRC8A	Proximity Label-MS	Samavarchi-Tehrani P (2020)	DOI:10.1101/2020.09.03.282103	-10.007
ORF7a	HCN2	Proximity Label-MS	Samavarchi-Tehrani P (2020)	DOI:10.1101/2020.09.03.282103	-10.467
ORF7a	SCN9A	Proximity Label-MS	Samavarchi-Tehrani P (2020)	DOI:10.1101/2020.09.03.282103	-10.087
ORF7a	VDAC3	Proximity Label-MS	Samavarchi-Tehrani P (2020)	DOI:10.1101/2020.09.03.282103	-10.095

ORF7a	GLRB	Proximity Label-MS	Laurent E (2020)	DOI:10.1101/2020.08.28.272955	-10.268
ORF7a	KCNJ11	Proximity Label-MS	Laurent E (2020)	DOI:10.1101/2020.08.28.272955	-10.173
ORF7a	LRRC8B	Proximity Label-MS	Laurent E (2020)	DOI:10.1101/2020.08.28.272955	-10.87
ORF7a	LRRC8D	Proximity Label-MS	St-Germain JR (2020)	DOI:10.1101/2020.08.28.269175	-10.625
ORF7a	TRPM7	Proximity Label-MS	St-Germain JR (2020)	DOI:10.1101/2020.08.28.269175	-10.87
ORF7a	CHRNA5	Proximity Label-MS	St-Germain JR (2020)	DOI:10.1101/2020.08.28.269175	-9.938
ORF7a	GJC1	Proximity Label-MS	St-Germain JR (2020)	DOI:10.1101/2020.08.28.269175	-9.347
ORF7a	KCNB2	Proximity Label-MS	St-Germain JR (2020)	DOI:10.1101/2020.08.28.269175	-10.87
ORF7a	LRRC8B	Proximity Label-MS	St-Germain JR (2020)	DOI:10.1101/2020.08.28.269175	-10.87
ORF7a	LRRC8C	Proximity Label-MS	St-Germain JR (2020)	DOI:10.1101/2020.08.28.269175	-10.295
ORF7b	ANO6	Affinity Capture-MS	Stukalov A (2020)	DOI:10.1101/2020.06.17.156455	-10.454
ORF7b	CACNG6	Affinity Capture-MS	Stukalov A (2020)	DOI:10.1101/2020.06.17.156455	-10.685
ORF7b	TRPM4	Affinity Capture-MS	Stukalov A (2020)	DOI:10.1101/2020.06.17.156455	-10.883
ORF7b	LRRC8A	Affinity Capture-MS	Stukalov A (2020)	DOI:10.1101/2020.06.17.156455	-10.264
ORF7b	GJA1	Affinity Capture-MS	Stukalov A (2020)	DOI:10.1101/2020.06.17.156455	-10.183
ORF7b	CLCN7	Proximity Label-MS	Samavarchi-Tehrani P	DOI:10.1101/2020.09.03.282103	-10.2

			(2020)		
ORF7b	GABRA5	Proximity Label-MS	Samavarchi-Tehrani P (2020)	DOI:10.1101/2020.09.03.282103	-10.707
ORF7b	ITPR1	Proximity Label-MS	Samavarchi-Tehrani P (2020)	DOI:10.1101/2020.09.03.282103	-10.157
ORF7b	ITPR2	Proximity Label-MS	Samavarchi-Tehrani P (2020)	DOI:10.1101/2020.09.03.282103	-10.475
ORF7b	ITPR3	Proximity Label-MS	Samavarchi-Tehrani P (2020)	DOI:10.1101/2020.09.03.282103	-10.373
ORF7b	KCNN4	Proximity Label-MS	Samavarchi-Tehrani P (2020)	DOI:10.1101/2020.09.03.282103	-10.389
ORF7b	PKD2	Proximity Label-MS	Samavarchi-Tehrani P (2020)	DOI:10.1101/2020.09.03.282103	-10.352
ORF7b	TPCN1	Proximity Label-MS	Samavarchi-Tehrani P (2020)	DOI:10.1101/2020.09.03.282103	-10.402
ORF7b	TRPM7	Proximity Label-MS	Samavarchi-Tehrani P (2020)	DOI:10.1101/2020.09.03.282103	-10.87
ORF7b	ANO10	Proximity Label-MS	Samavarchi-Tehrani P (2020)	DOI:10.1101/2020.09.03.282103	-10.066
ORF7b	LRRC8D	Proximity Label-MS	Samavarchi-Tehrani P (2020)	DOI:10.1101/2020.09.03.282103	-10.866
ORF7b	ANO8	Proximity Label-MS	Samavarchi-Tehrani P (2020)	DOI:10.1101/2020.09.03.282103	-10.398

ORF7b	HCN2	Proximity Label-MS	Samavarchi-Tehrani P (2020)	DOI:10.1101/2020.09.03.282103	-10.731
ORF7b	VDAC3	Proximity Label-MS	Samavarchi-Tehrani P (2020)	DOI:10.1101/2020.09.03.282103	-10.636
ORF7b	CLCN2	Proximity Label-MS	Laurent E (2020)	DOI:10.1101/2020.08.28.272955	-10.598
ORF7b	GRID1	Proximity Label-MS	Laurent E (2020)	DOI:10.1101/2020.08.28.272955	-10.823
ORF7b	LRRC8B	Proximity Label-MS	Laurent E (2020)	DOI:10.1101/2020.08.28.272955	-10.87
ORF7b	ASIC1	Proximity Label-MS	St-Germain JR (2020)	DOI:10.1101/2020.08.28.269175	-10.846
ORF7b	LRRC8C	Proximity Label-MS	St-Germain JR (2020)	DOI:10.1101/2020.08.28.269175	-10.547
ORF7b	LRRC8E	Proximity Label-MS	St-Germain JR (2020)	DOI:10.1101/2020.08.28.269175	-10.87
ORF8	ANO6	Proximity Label-MS	Samavarchi-Tehrani P (2020)	DOI:10.1101/2020.09.03.282103	-10.87
ORF8	TRPM4	Proximity Label-MS	Samavarchi-Tehrani P (2020)	DOI:10.1101/2020.09.03.282103	-10.87
ORF8	LRRC8D	Proximity Label-MS	Samavarchi-Tehrani P (2020)	DOI:10.1101/2020.09.03.282103	-10.87
ORF8	CACNA2D1	Proximity Label-MS	Samavarchi-Tehrani P (2020)	DOI:10.1101/2020.09.03.282103	-10.87
ORF8	LRRC8E	Proximity Label-MS	Samavarchi-Tehrani P (2020)	DOI:10.1101/2020.09.03.282103	-10.894

ORF8	LRRC8A	Proximity Label-MS	Laurent E (2020)	DOI:10.1101/2020.08.28.272955	-9.884
ORF8	ANO10	Proximity Label-MS	St-Germain JR (2020)	DOI:10.1101/2020.08.28.269175	-10.402
ORF8	ASIC1	Proximity Label-MS	St-Germain JR (2020)	DOI:10.1101/2020.08.28.269175	-10.87
ORF8	CLCN7	Proximity Label-MS	St-Germain JR (2020)	DOI:10.1101/2020.08.28.269175	-10.843
ORF8	ITPR2	Proximity Label-MS	St-Germain JR (2020)	DOI:10.1101/2020.08.28.269175	-10.87
ORF8	ITPR3	Proximity Label-MS	St-Germain JR (2020)	DOI:10.1101/2020.08.28.269175	-10.87
ORF8	KCNB2	Proximity Label-MS	St-Germain JR (2020)	DOI:10.1101/2020.08.28.269175	-10.87
ORF8	LRRC8C	Proximity Label-MS	St-Germain JR (2020)	DOI:10.1101/2020.08.28.269175	-10.232
ORF8	MCOLN3	Proximity Label-MS	St-Germain JR (2020)	DOI:10.1101/2020.08.28.269175	-10.87
ORF8	TRPM7	Proximity Label-MS	St-Germain JR (2020)	DOI:10.1101/2020.08.28.269175	-10.87
nsp3	KCNJ11	Proximity Label-MS	St-Germain JR (2020)	DOI:10.1101/2020.08.28.269175	-11.235
nsp4	ANO6	Proximity Label-MS	Samavarchi-Tehrani P (2020)	DOI:10.1101/2020.09.03.282103	-10.605
nsp4	GABRA5	Proximity Label-MS	Samavarchi-Tehrani P (2020)	DOI:10.1101/2020.09.03.282103	-10.945
nsp4	GJA1	Proximity Label-MS	Samavarchi-Tehrani P (2020)	DOI:10.1101/2020.09.03.282103	-10.518
nsp4	ITPR1	Proximity	Samavarchi-Tehrani P	DOI:10.1101/20	-10.205

		Label-MS	(2020)	20.09.03.282103	
nsp4	ITPR2	Proximity Label-MS	Samavarchi-Tehrani P (2020)	DOI:10.1101/2020.09.03.282103	-10.523
nsp4	ITPR3	Proximity Label-MS	Samavarchi-Tehrani P (2020)	DOI:10.1101/2020.09.03.282103	-10.424
nsp4	KCNN4	Proximity Label-MS	Samavarchi-Tehrani P (2020)	DOI:10.1101/2020.09.03.282103	-10.673
nsp4	PKD2	Proximity Label-MS	Samavarchi-Tehrani P (2020)	DOI:10.1101/2020.09.03.282103	-10.503
nsp4	TRPM7	Proximity Label-MS	Samavarchi-Tehrani P (2020)	DOI:10.1101/2020.09.03.282103	-10.87
nsp4	ANO10	Proximity Label-MS	Samavarchi-Tehrani P (2020)	DOI:10.1101/2020.09.03.282103	-10.309
nsp4	LRRC8D	Proximity Label-MS	Samavarchi-Tehrani P (2020)	DOI:10.1101/2020.09.03.282103	-10.992
nsp4	VDAC2	Proximity Label-MS	Samavarchi-Tehrani P (2020)	DOI:10.1101/2020.09.03.282103	-11.142
nsp4	VDAC3	Proximity Label-MS	Samavarchi-Tehrani P (2020)	DOI:10.1101/2020.09.03.282103	-10.93
nsp4	TRPA1	Proximity Label-MS	Samavarchi-Tehrani P (2020)	DOI:10.1101/2020.09.03.282103	-10.302
nsp4	ANO5	Proximity Label-MS	Laurent E (2020)	DOI:10.1101/2020.08.28.272955	-10.141

nsp4	CLCN2	Proximity Label-MS	Laurent E (2020)	DOI:10.1101/2020.08.28.272955	-10.743
nsp4	GRID1	Proximity Label-MS	Laurent E (2020)	DOI:10.1101/2020.08.28.272955	-10.946
nsp4	KCNQ5	Proximity Label-MS	Laurent E (2020)	DOI:10.1101/2020.08.28.272955	-10.87
nsp4	LRRC8B	Proximity Label-MS	Laurent E (2020)	DOI:10.1101/2020.08.28.272955	-10.87
nsp4	TRPM4	Proximity Label-MS	Laurent E (2020)	DOI:10.1101/2020.08.28.272955	-10.984
nsp4	ASIC1	Proximity Label-MS	St-Germain JR (2020)	DOI:10.1101/2020.08.28.269175	-11.012
nsp4	CLCN7	Proximity Label-MS	St-Germain JR (2020)	DOI:10.1101/2020.08.28.269175	-10.393
nsp4	KCNB2	Proximity Label-MS	St-Germain JR (2020)	DOI:10.1101/2020.08.28.269175	-10.87
nsp4	LRRC8A	Proximity Label-MS	St-Germain JR (2020)	DOI:10.1101/2020.08.28.269175	-10.444
nsp4	LRRC8C	Proximity Label-MS	St-Germain JR (2020)	DOI:10.1101/2020.08.28.269175	-10.702
nsp4	LRRC8E	Proximity Label-MS	St-Germain JR (2020)	DOI:10.1101/2020.08.28.269175	-10.87
nsp5	VDAC3	Proximity Label-MS	Samavarchi-Tehrani P (2020)	DOI:10.1101/2020.09.03.282103	-11.575
nsp6	GJC1	Proximity Label-MS	Samavarchi-Tehrani P (2020)	DOI:10.1101/2020.09.03.282103	-10.619
nsp6	CLCN7	Proximity Label-MS	Samavarchi-Tehrani P (2020)	DOI:10.1101/2020.09.03.282103	-10.752
nsp6	ANO6	Proximity	Samavarchi-Tehrani P	DOI:10.1101/20	-10.17

		Label-MS	(2020)	20.09.03.282103	
nsp6	GABRA5	Proximity Label-MS	Samavarchi-Tehrani P (2020)	DOI:10.1101/2020.09.03.282103	-10.678
nsp6	GJA1	Proximity Label-MS	Samavarchi-Tehrani P (2020)	DOI:10.1101/2020.09.03.282103	-10.588
nsp6	ITPR2	Proximity Label-MS	Samavarchi-Tehrani P (2020)	DOI:10.1101/2020.09.03.282103	-10.87
nsp6	ITPR3	Proximity Label-MS	Samavarchi-Tehrani P (2020)	DOI:10.1101/2020.09.03.282103	-10.87
nsp6	KCNN4	Proximity Label-MS	Samavarchi-Tehrani P (2020)	DOI:10.1101/2020.09.03.282103	-11.479
nsp6	PKD2	Proximity Label-MS	Samavarchi-Tehrani P (2020)	DOI:10.1101/2020.09.03.282103	-9.868
nsp6	TRPM4	Proximity Label-MS	Samavarchi-Tehrani P (2020)	DOI:10.1101/2020.09.03.282103	-10.87
nsp6	ANO10	Proximity Label-MS	Samavarchi-Tehrani P (2020)	DOI:10.1101/2020.09.03.282103	-10.349
nsp6	HCN2	Proximity Label-MS	Samavarchi-Tehrani P (2020)	DOI:10.1101/2020.09.03.282103	-10.87
nsp6	VDAC2	Proximity Label-MS	Samavarchi-Tehrani P (2020)	DOI:10.1101/2020.09.03.282103	-10.946
nsp6	VDAC3	Proximity Label-MS	Samavarchi-Tehrani P (2020)	DOI:10.1101/2020.09.03.282103	-10.933

nsp6	LRRC8A	Proximity Label-MS	Laurent E (2020)	DOI:10.1101/2020.08.28.272955	-9.868
nsp6	ASIC1	Proximity Label-MS	St-Germain JR (2020)	DOI:10.1101/2020.08.28.269175	-10.87
nsp6	KCNB2	Proximity Label-MS	St-Germain JR (2020)	DOI:10.1101/2020.08.28.269175	-10.87
nsp6	LRRC8C	Proximity Label-MS	St-Germain JR (2020)	DOI:10.1101/2020.08.28.269175	-10.192
nsp6	LRRC8D	Proximity Label-MS	St-Germain JR (2020)	DOI:10.1101/2020.08.28.269175	-10.87
nsp6	TRPM7	Proximity Label-MS	St-Germain JR (2020)	DOI:10.1101/2020.08.28.269175	-10.87
nsp13	VDAC3	Proximity Label-MS	Samavarchi-Tehrani P (2020)	DOI:10.1101/2020.09.03.282103	-10.87
nsp14	VDAC3	Proximity Label-MS	Samavarchi-Tehrani P (2020)	DOI:10.1101/2020.09.03.282103	-10.801
nsp16	CLIC1	Proximity Label-MS	Laurent E (2020)	DOI:10.1101/2020.08.28.272955	-10.3
N	E	Two-hybrid	Liang Q (2020)	DOI:10.1101/2020.03.31.019216	-10.897
nsp10	nsp16	Co-crystal Structure	Wilamowski M (2020)	DOI:10.1101/2020.08.14.251421	-11.686
nsp12	nsp7	Co-crystal Structure	Gao Y (2020)	DOI:10.1101/2020.03.16.993386	-11.117
nsp12	nsp8	Co-crystal Structure	Gao Y (2020)	DOI:10.1101/2020.03.16.993386	-10.911
nsp14	nsp10	Two-hybrid	Liang Q (2020)	DOI:10.1101/2020.03.31.019216	-10.87
nsp14	nsp10	Co-purification	Baddock HT (2020)	DOI:10.1101/2020.08.13.248211	-10.87

nsp15	nsp2	Two-hybrid	Liang Q (2020)	DOI:10.1101/2020.03.31.019216	-10.87
nsp7	nsp9	Two-hybrid	Liang Q (2020)	DOI:10.1101/2020.03.31.019216	-10.87
nsp7	ORF7a	Two-hybrid	Liang Q (2020)	DOI:10.1101/2020.03.31.019216	-11.372
nsp7	nsp8	Co-crystal Structure	Shi W (2020)	DOI:10.1101/2020.07.30.229187	-11.122
nsp8	ORF8	Two-hybrid	Liang Q (2020)	DOI:10.1101/2020.03.31.019216	-10.87
ORF3a	E	Two-hybrid	Liang Q (2020)	DOI:10.1101/2020.03.31.019216	-10.932
ORF6	nsp14	Two-hybrid	Liang Q (2020)	DOI:10.1101/2020.03.31.019216	-10.87
ORF6	nsp3	Affinity Capture-MS	Stukalov A (2020)	DOI:10.1101/2020.06.17.156455	-11.154
ORF7a	N	Affinity Capture-MS	Stukalov A (2020)	DOI:10.1101/2020.06.17.156455	-10.595
ORF7b	nsp1	Two-hybrid	Liang Q (2020)	DOI:10.1101/2020.03.31.019216	-10.87
ORF7b	nsp7	Two-hybrid	Liang Q (2020)	DOI:10.1101/2020.03.31.019216	-11.072

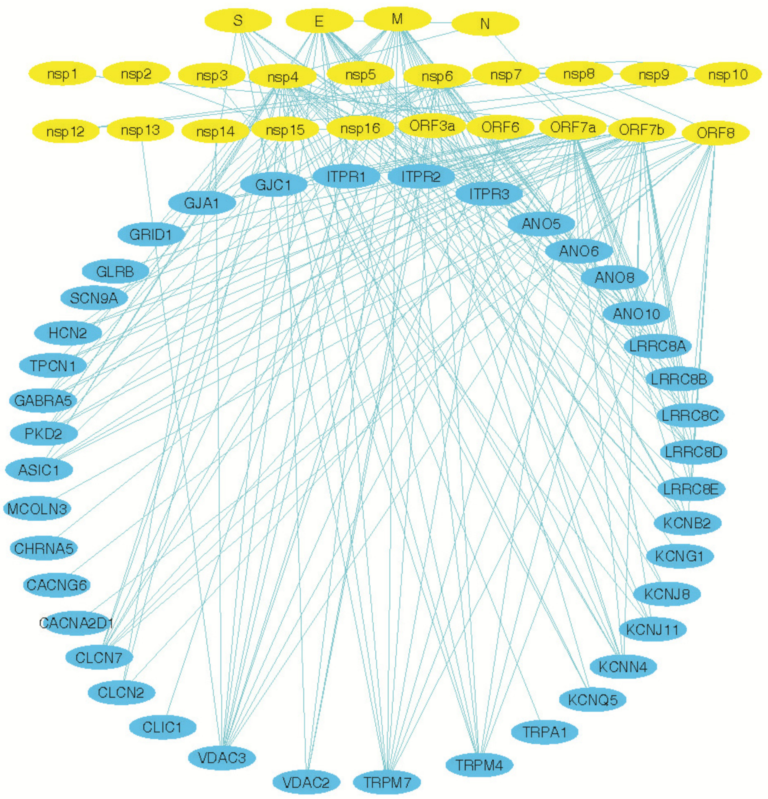

Figure S1. Schematic depiction of interactions of total SARS-CoV-2 proteins with human ion channels

Table S2: Function of human ion channels interacting with SARS-CoV-2 proteins

	Protein	Protein name	Function	References
1	ITPR1	inositol 1,4,5-trisphosphate receptor type 1	Calcium transportation from endoplasmic reticulum	[1]
2	ITPR2	inositol 1,4,5-trisphosphate receptor type 2	Calcium transportation from endoplasmic reticulum	[2,3]
3	ITPR3	inositol 1,4,5-trisphosphate receptor type 3	Calcium transportation from endoplasmic reticulum	[4]

4	ANO5	anoctamin 5	Transmembrane protein, it's a calcium activated chloride channel	[5]
5	ANO6	anoctamin 6	Essential component for the calcium-dependent exposure of phosphatidylserine on the cell surface	[6-8]
6	ANO8	anoctamin 8	Membrane contact sites formation in the ER/PM junctions	[9]
7	ANO10	anoctamin 10	Transmembrane protein, it's a calcium activated chloride channel	[10,11]
8	LRRC8A	leucine rich repeat containing 8 VRAC subunit A	Four-pass transmembrane protein that plays a role in B cell development	[12,13]
9	LRRC8B	leucine rich repeat containing 8 VRAC subunit B	Anion channel required to maintain a constant cell volume in response to extracellular or intracellular osmotic changes	[14-16]
10	LRRC8C	leucine rich repeat containing 8 VRAC subunit C	Plays a redundant role in the efflux of amino acids, such as aspartate and glutamate, in response to osmotic	[14-16]

			stress. Channel activity requires LRRC8A plus at least one other family member (LRRC8B, LRRC8C, LRRC8D or LRRC8E)	
11	LRRC8D	leucine rich repeat containing 8 VRAC subunit D	Mediates the import of the antibiotic blasticidin-S into the cell	[17]
12	LRRC8E	leucine rich repeat containing 8 VRAC subunit E	Anion channel required to maintain a constant cell volume in response to extracellular or intracellular osmotic changes	[18]
13	KCNB2	potassium voltage-gated channel subfamily B member 2	Delayed rectifier potassium channel; functions include regulating neurotransmitter release, heart rate, insulin secretion, epithelial electrolyte transport, and cell volume.	[19,20]
14	KCNG1	potassium voltage-gated channel modifier subfamily G member 1	Form functional heterotetrameric channels with KCNB1; modulates the delayed rectifier voltage-gated	[21]

			potassium channel activation and deactivation rates of KCNB1	
15	KCNJ8	potassium voltage-gated channel subfamily J member 8	Protein has a greater tendency to allow potassium to flow into a cell rather than out of a cell	[22,23]
16	KCNJ11	potassium voltage-gated channel subfamily J member 11	Subunit of ATP-sensitive potassium channels; form cardiac and smooth muscle-type KATP channels with ABCC9. KCNJ11 forms the channel pore while ABCC9 is required for activation and regulation	[24-26]
17	KCNN4	potassium calcium-activated channel subfamily N member 4	Required for maximal calcium influx and proliferation during the reactivation of naive T-cells. Plays a role in the late stages of EGF-induced macropinocytosis	[27-29]
18	KCNQ5	potassium voltage-gated channel subfamily Q member 5	Protein yields currents that activate slowly with depolarization and can form heteromeric	[30]

			channels with the protein encoded by the KCNQ3 gene	
19	TRPA1	transient receptor potential cation channel subfamily A member 1	May involve a role in signal transduction and growth control	[31]
20	TRPM4	transient receptor potential cation channel subfamily M member 4	Calcium-activated nonselective ion channel that mediates transport of monovalent cations across membranes, thereby depolarizing the membrane	[32,33]
21	TRPM7	transient receptor potential cation channel subfamily M member 7	Protein is both, an ion channel and a serine/threonine protein kinase. The kinase activity is essential for the ion channel function, which serves to increase intracellular calcium levels and to help regulate magnesium ion homeostasis.	[34]
22	VDAC2	voltage dependent anion channel 2	Involvement of Ca^{+2} in viral entry	[35,36]
23	VDAC3	voltage dependent anion channel 3	Involvement of Ca^{+2} in viral entry	[37]

24	CLIC1	chloride voltage-gated channel 1	Protein localizes principally to the cell nucleus and exhibits both nuclear and plasma membrane chloride ion channel activity	[38]
25	CLCN2	chloride voltage-gated channel 2	Transmembrane protein that maintains chloride ion homeostasis in various cells	[39]
26	CLCN7	chloride voltage-gated channel 7	Functions as antiporter and contributes to the acidification of the lysosome lumen and may be involved in maintaining lysosomal pH	[40]
27	CACNA2D1	calcium voltage-gated channel auxiliary subunit alpha2delta 1	Calcium channels mediate the influx of calcium ions into the cell upon membrane polarization	[41]
28	CACNG6	calcium voltage-gated channel auxiliary subunit gamma 6	Regulates the activity of L-type calcium channels that contain CACNA1C as pore-forming subunit	[42]
29	GJA1	gap junction protein alpha 1	Protein is a component of gap junctions, composed of arrays	[43,44]

			of intercellular channels that provide a route for the diffusion of low molecular weight materials from cell to cell. Major protein of gap junctions in the heart that have crucial role in the synchronized contraction of the heart	
30	GJC1	gap junction protein gamma 1	Protein is a component of gap junctions, composed of arrays of intercellular channels that provide a route for the diffusion of low molecular weight materials from cell to cell. Major protein of gap junctions in the heart that have crucial role in the synchronized contraction of the heart	[45]
31	ASIC1	acid sensing ion channel subunit 1	Part of the degenerin/epithelial sodium channel (DEG/ENaC) superfamily. Proteins function in learning, pain	[46]

			transduction, touch sensation, and development of memory and fear	
32	PKD2	polycystin 2, transient receptor potential cation channel	Multi-pass membrane protein that functions as a calcium permeable cation channel	[47]
33	GABRA5	gamma-aminobutyric acid type A receptor alpha5 subunit	Ligand-gated chloride channel subunit which is a component of the heteropentameric receptor for GABA; may be involved in GABA-A receptor assembly	[48]
34	TPCN1	two pore segment channel 1	Function as one of the major voltage-gated Ca2+ channels (VDCC) across the lysosomal and endosomal membrane	[49]
35	HCN2	hyperpolarization activated cyclic nucleotide gated potassium and sodium channel 2	Ion channel exhibiting weak selectivity for potassium over sodium ions. Contributes to the native pacemaker currents in heart	[50]
36	SCN9A	sodium voltage-gated channel alpha subunit 9	Protein forms a sodium-selective channel through	[51]

			which Na+ ions may pass in accordance with their electrochemical gradient	
37	GLRB	glycine receptor beta	GLRB does not form ligand-gated ion channels by itself, but is part of heteromeric ligand-gated chloride channels	[52]
38	GRID1	glutamate ionotropic receptor delta type subunit 1	Subunit of glutamate receptor channels; play key roles in synaptic plasticity	[48]
39	CHRNA5	cholinergic receptor nicotinic alpha 5 subunit	Protein subunit is a member of a superfamily of ligand-gated ion channels that mediate fast signal transmission at synapses	[53]
40	MCOLN3	mucolipin 3	Acts as Ca2+-permeable cation channel with inwardly rectifying activity	[54]

Table S3: A list of drugs interacting with HICs

	Compound Name	Compound Structure	Human Protein	Viral Protein
1	Adenosine Diphosphate	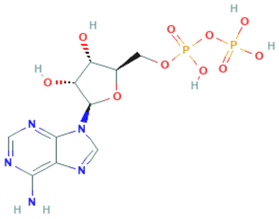	TRPM4	ORF8,ORF14, M, E, S, ORF7a, ORF7b, nsp4, nsp6,
2	Clotrimazole	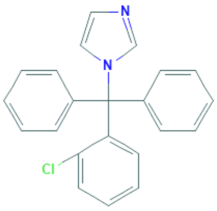		
3	Adenosine	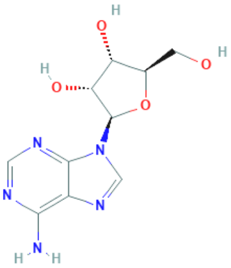		
4	Adenosine Triphosphate	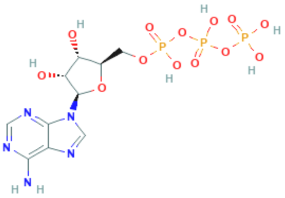		
5	Glyburide	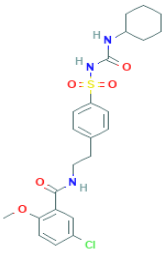		

6	Spermine	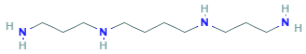		
7	Thymol	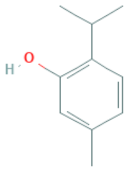	TRPA1	nsp4
8	Benzoquinone	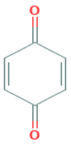		
9	Chloropicrin	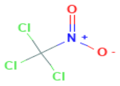		
10	Allicin	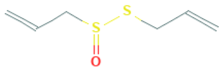		

11	Morphanthridine	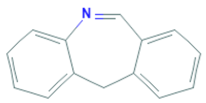		
12	Polygodial	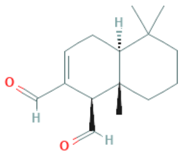		
13	Methylglyoxal	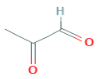		
14	Isovelleral	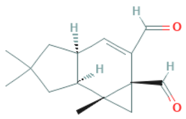		
15	Acrolein	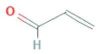		
16	Nicotine	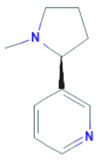		

17	Menthol	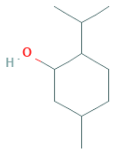		
18	Acetaldehyde	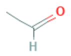		
19	Salirasib	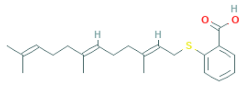		
20	Auranofin	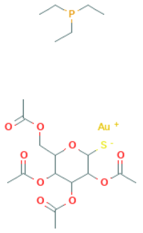		
21	Apomorphine	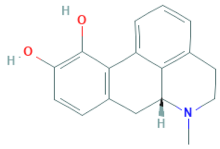		
22	Cannabidivarin	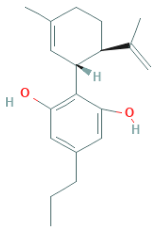		

23	Tetrahydrocannabivarin	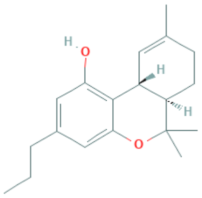		
24	Levomenthol	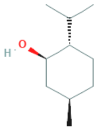		
25	Butamben	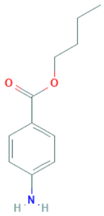		
26	Camphor	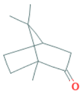		
27	Cannabidiol	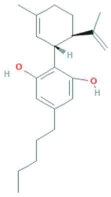		
28	Nabiximols	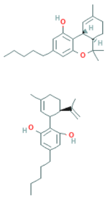		
29	Phenethylisothiocyanate	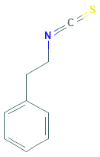		

30	Benzyl isothiocyanate	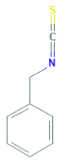		
31	Isopropyl isothiocyanate	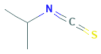		
32	Voacangine	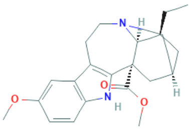		
33	Erucin	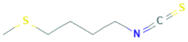		
34	Allyl isothiocyanate	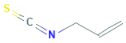		
35	4-Hydroxynon-2-enal	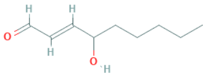		

36	Carbenoxolone	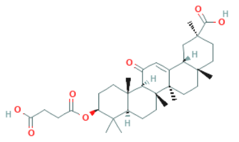	GJA1	M, ORF7a, ORF7b, nsp4, nsp6, ORF14, ORF3b,
37	Octanol	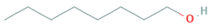		
38	Carvedilol	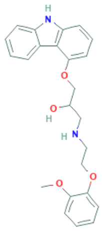		
39	Epigalocatechin Gallate	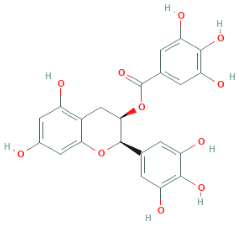		
40	Bleomycin	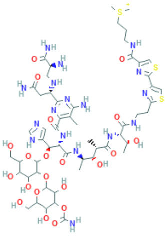		
41	Propylthiouracil	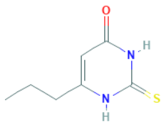		

42	Labetalol	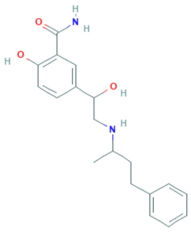		
43	Atenolol	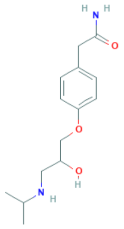		
44	Chlorzoxazone	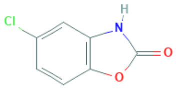	KCNN4	M, E, S, ORF7a, ORF7b, nsp4, nsp6,
45	Senicapoc	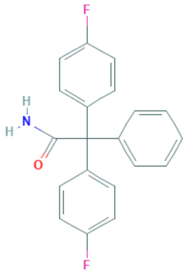		
46	Clotrimazole	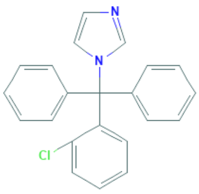		
47	Nitredipine	NA		

48	Riluzole	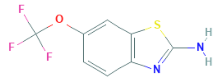		
49	Quinine	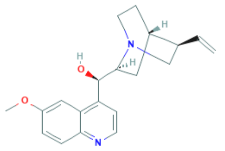		
50	Halothane	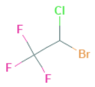		
51	Diminazene	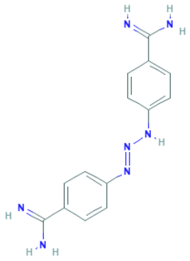	ASIC1	E, ORF7a, ORF7b, nsp4, nsp6, ORF8,
52	Amiloride	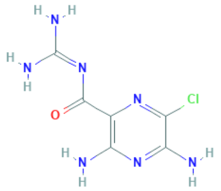		
53	Ibuprofen	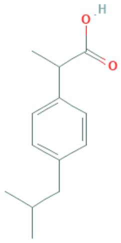		

54	Nafamostat	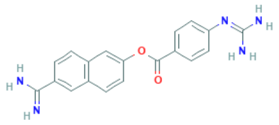		
55	Benzamil	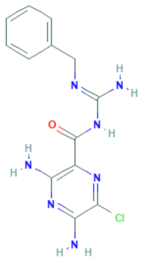		
56	Caffeine	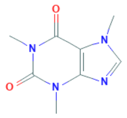	ITPR1	M, E, ORF7a, ORF7b, nsp4,
57	Adenosine Triphosphate	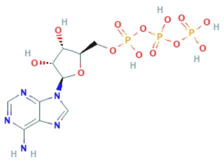		
58	Nitroprusside	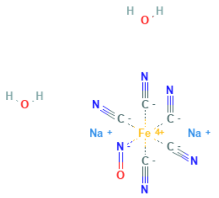		
59	Glycerin	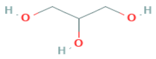		

References

1. Atakpa, P.; Thillaiappan, N.B.; Mataragka, S.; Prole, D.L.; Taylor, C.W. IP3 Receptors Preferentially Associate with ER-Lysosome Contact Sites and Selectively Deliver Ca(2+) to Lysosomes. *Cell Rep* **2018**, *25*, 3180-3193 e3187, doi:10.1016/j.celrep.2018.11.064.
2. Wiel, C.; Lallet-Daher, H.; Gitenay, D.; Gras, B.; Le Calve, B.; Augert, A.; Ferrand, M.; Prevarskaya, N.; Simonnet, H.; Vindrieux, D.; et al. Endoplasmic reticulum calcium release through ITPR2 channels leads to mitochondrial calcium accumulation and senescence. *Nat Commun* **2014**, *5*, 3792, doi:10.1038/ncomms4792.
3. Vervloessem, T.; Yule, D.I.; Bultynck, G.; Parys, J.B. The type 2 inositol 1,4,5-trisphosphate receptor, emerging functions for an intriguing Ca(2)(+)-release channel. *Biochim Biophys Acta* **2015**, *1853*, 1992-2005, doi:10.1016/j.bbamcr.2014.12.006.
4. Kuchay, S.; Saeed, M.; Giorgi, C.; Li, J.; Hoffmann, H.H.; Pinton, P.; Rice, C.M.; Pagano, M. NS5A Promotes Constitutive Degradation of IP3R3 to Counteract Apoptosis Induced by Hepatitis C Virus. *Cell Rep* **2018**, *25*, 833-840 e833, doi:10.1016/j.celrep.2018.09.088.
5. Oh, U.; Jung, J. Cellular functions of TMEM16/anoctamin. *Pflugers Arch* **2016**, *468*, 443-453, doi:10.1007/s00424-016-1790-0
6. Lin, H.; Jun, I.; Woo, J.H.; Lee, M.G.; Kim, S.J.; Nam, J.H. Temperature-dependent increase in the calcium sensitivity and acceleration of activation of ANO6 chloride channel variants. *Sci Rep* **2019**, *9*, 6706, doi:10.1038/s41598-019-43162-1
7. Veit, M.; Koyro, K.I.; Ahrens, B.; Bleibaum, F.; Munz, M.; Rovekamp, H.; Andra, J.; Schreiber, R.; Kunzelmann, K.; Sommer, A.; et al. Anoctamin-6 regulates ADAM sheddase function. *Biochim Biophys Acta Mol Cell Res* **2018**, *1865*, 1598-1610, doi:10.1016/j.bbamcr.2018.08.011.
8. Lin, H.; Roh, J.; Woo, J.H.; Kim, S.J.; Nam, J.H. TMEM16F/ANO6, a Ca(2+)-activated anion channel, is negatively regulated by the actin cytoskeleton and intracellular MgATP. *Biochem Biophys Res Commun* **2018**, *503*, 2348-2354, doi:10.1016/j.bbrc.2018.06.160.
9. Jha, A.; Chung, W.Y.; Vachel, L.; Maleth, J.; Lake, S.; Zhang, G.; Ahuja, M.; Muallem, S. Anoctamin 8 tethers endoplasmic reticulum and plasma membrane for assembly of Ca(2+) signaling complexes at the ER/PM compartment. *EMBO J* **2019**, *38*, doi:10.15252/embj.2018101452.
10. Bushell, S.R.; Pike, A.C.W.; Falzone, M.E.; Rorsman, N.J.G.; Ta, C.M.; Corey, R.A.; Newport, T.D.; Christianson, J.C.; Scofano, L.F.; Shintre, C.A.; et al. The structural basis of lipid scrambling and inactivation in the endoplasmic reticulum scramblase TMEM16K. *Nat Commun* **2019**, *10*, 3956, doi:10.1038/s41467-019-11753-1
11. Ishihara, K.; Suzuki, J.; Nagata, S. Role of Ca(2+) in the Stability and Function of TMEM16F and 16K. *Biochemistry* **2016**, *55*, 3180-3188, doi:10.1021/acs.biochem.6b00176.
12. Chen, L.; Konig, B.; Liu, T.; Pervaiz, S.; Razzaque, Y.S.; Stauber, T. More than just a pressure relief valve: physiological roles of volume-regulated LRRC8 anion channels. *Biol Chem* **2019**, *400*, 1481-1496, doi:10.1515/hsz-2019-0189
13. Lu, J.; Xu, F.; Zhang, J. Inhibition of angiotensin II-induced cerebrovascular smooth muscle cell proliferation by LRRC8A downregulation through suppressing PI3K/AKT activation. *Hum Cell* **2019**, *32*, 316-325, doi:10.1007/s13577-019-00260-6
14. Voss, F.K.; Ullrich, F.; Munch, J.; Lazarow, K.; Lutter, D.; Mah, N.; Andrade-Navarro, M.A.; von Kries, J.P.; Stauber, T.; Jentsch, T.J. Identification of LRRC8 heteromers as an essential component of the volume-regulated anion channel VRAC. *Science* **2014**, *344*, 634-638, doi:10.1126/science.1252826.
15. Syeda, R.; Qiu, Z.; Dubin, A.E.; Murthy, S.E.; Florendo, M.N.; Mason, D.E.; Mathur, J.; Cahalan, S.M.; Peters, E.C.; Montal, M.; et al. LRRC8 Proteins Form Volume-Regulated Anion Channels that Sense Ionic Strength. *Cell* **2016**, *164*, 499-511, doi:10.1016/j.cell.2015.12.031.
16. Lutter, D.; Ullrich, F.; Lueck, J.C.; Kempa, S.; Jentsch, T.J. Selective transport of neurotransmitters and modulators by distinct volume-regulated LRRC8 anion channels. *J Cell Sci* **2017**, *130*, 1122-1133, doi:10.1242/jcs.196253.

17. Lee, C.C.; Freinkman, E.; Sabatini, D.M.; Ploegh, H.L. The protein synthesis inhibitor blasticidin s enters mammalian cells via leucine-rich repeat-containing protein 8D. *J Biol Chem* **2014**, *289*, 17124-17131, doi:10.1074/jbc.M114.571257.
18. Schober, A.L.; Wilson, C.S.; Mongin, A.A. Molecular composition and heterogeneity of the LRRC8-containing swelling-activated osmolyte channels in primary rat astrocytes. *J Physiol* **2017**, *595*, 6939-6951, doi:10.1113/JP275053.
19. Fu, J.; Dai, X.; Plummer, G.; Suzuki, K.; Bautista, A.; Githaka, J.M.; Senior, L.; Jensen, M.; Greitzer-Antes, D.; Manning Fox, J.E.; et al. Kv2.1 Clustering Contributes to Insulin Exocytosis and Rescues Human beta-Cell Dysfunction. *Diabetes* **2017**, *66*, 1890-1900, doi:10.2337/db16-1170.
20. Yan, L.; Figueroa, D.J.; Austin, C.P.; Liu, Y.; Bugianesi, R.M.; Slaughter, R.S.; Kaczorowski, G.J.; Kohler, M.G. Expression of voltage-gated potassium channels in human and rhesus pancreatic islets. *Diabetes* **2004**, *53*, 597-607, doi:10.2337/diabetes.53.3.597.
21. Mederos, Y.S.M.; Rinne, S.; Skrobek, L.; Renigunta, V.; Schlichthorl, G.; Derst, C.; Gudermann, T.; Daut, J.; Preisig-Muller, R. Mutation of histidine 105 in the T1 domain of the potassium channel Kv2.1 disrupts heteromerization with Kv6.3 and Kv6.4. *J Biol Chem* **2009**, *284*, 4695-4704, doi:10.1074/jbc.M808786200.
22. Du, Q.; Jovanovic, S.; Tulic, L.; Sljivancanin, D.; Jack, D.W.; Zizic, V.; Abdul, K.S.; Tulic, I.; Jovanovic, A. KATP channels are up-regulated with increasing age in human myometrium. *Mech Ageing Dev* **2013**, *134*, 98-102, doi:10.1016/j.mad.2013.01.003.
23. Delaney, J.T.; Muhammad, R.; Blair, M.A.; Kor, K.; Fish, F.A.; Roden, D.M.; Darbar, D. A KCNJ8 mutation associated with early repolarization and atrial fibrillation. *Europace* **2012**, *14*, 1428-1432, doi:10.1093/europace/eus150.
24. Babenko, A.P.; Gonzalez, G.; Aguilar-Bryan, L.; Bryan, J. Reconstituted human cardiac KATP channels: functional identity with the native channels from the sarcolemma of human ventricular cells. *Circ Res* **1998**, *83*, 1132-1143, doi:10.1161/01.res.83.11.1132.
25. Tammaro, P.; Ashcroft, F.M. A mutation in the ATP-binding site of the Kir6.2 subunit of the KATP channel alters coupling with the SUR2A subunit. *J Physiol* **2007**, *584*, 743-753, doi:10.1113/jphysiol.2007.143149.
26. Cooper, P.E.; McClenaghan, C.; Chen, X.; Stary-Weinzinger, A.; Nichols, C.G. Conserved functional consequences of disease-associated mutations in the slide helix of Kir6.1 and Kir6.2 subunits of the ATP-sensitive potassium channel. *J Biol Chem* **2017**, *292*, 17387-17398, doi:10.1074/jbc.M117.804971.
27. Srivastava, S.; Li, Z.; Ko, K.; Choudhury, P.; Albaqumi, M.; Johnson, A.K.; Yan, Y.; Backer, J.M.; Unutmaz, D.; Coetzee, W.A.; et al. Histidine phosphorylation of the potassium channel KCa3.1 by nucleoside diphosphate kinase B is required for activation of KCa3.1 and CD4 T cells. *Mol Cell* **2006**, *24*, 665-675, doi:10.1016/j.molcel.2006.11.012.
28. Srivastava, S.; Zhdanova, O.; Di, L.; Li, Z.; Albaqumi, M.; Wulff, H.; Skolnik, E.Y. Protein histidine phosphatase 1 negatively regulates CD4 T cells by inhibiting the K+ channel KCa3.1. *Proc Natl Acad Sci U S A* **2008**, *105*, 14442-14446, doi:10.1073/pnas.0803678105.
29. Maekawa, M.; Terasaka, S.; Mochizuki, Y.; Kawai, K.; Ikeda, Y.; Araki, N.; Skolnik, E.Y.; Taguchi, T.; Arai, H. Sequential breakdown of 3-phosphorylated phosphoinositides is essential for the completion of macropinocytosis. *Proc Natl Acad Sci U S A* **2014**, *111*, E978-987, doi:10.1073/pnas.1311029111.
30. Zhang, Y.; Chu, X.; Liu, L.; Zhang, N.; Guo, H.; Yang, F.; Liu, Z.; Dong, Y.; Bao, Y.; Zhang, X.; et al. Tannic acid activates the Kv7.4 and Kv7.3/7.5 K(+) channels expressed in HEK293 cells and reduces tension in the rat mesenteric arteries. *J Pharm Pharmacol* **2016**, *68*, 494-502, doi:10.1111/jphp.12527.
31. Zhao, J.; Lin King, J.V.; Paulsen, C.E.; Cheng, Y.; Julius, D. Irritant-evoked activation and calcium modulation of the TRPA1 receptor. *Nature* **2020**, *585*, 141-145, doi:10.1038/s41586-020-2480-9

32. Xian, W.; Wang, H.; Moretti, A.; Laugwitz, K.L.; Flockerzi, V.; Lipp, P. Domain zipping and unzipping modulates TRPM4's properties in human cardiac conduction disease. *FASEB J* **2020**, *34*, 12114-12126, doi:10.1096/fj.202000097RR.
33. Jha, R.M.; Desai, S.M.; Zusman, B.E.; Koleck, T.A.; Puccio, A.M.; Okonkwo, D.O.; Park, S.Y.; Shutter, L.A.; Kochanek, P.M.; Conley, Y.P. Downstream TRPM4 Polymorphisms Are Associated with Intracranial Hypertension and Statistically Interact with ABCC8 Polymorphisms in a Prospective Cohort of Severe Traumatic Brain Injury. *J Neurotrauma* **2019**, *36*, 1804-1817, doi:10.1089/neu.2018.6124.
34. Lee, C.T.; Ng, H.Y.; Kuo, W.H.; Tain, Y.L.; Leung, F.F.; Lee, Y.T. The role of TRPM7 in vascular calcification: Comparison between phosphate and uremic toxin. *Life Sci* **2020**, *260*, 118280, doi:10.1016/j.lfs.2020.118280.
35. Luzio, J.P.; Bright, N.A.; Pryor, P.R. The role of calcium and other ions in sorting and delivery in the late endocytic pathway. *Biochem Soc Trans* **2007**, *35*, 1088-1091, doi:10.1042/BST0351088.
36. Fujioka, Y.; Nishide, S.; Ose, T.; Suzuki, T.; Kato, I.; Fukuhara, H.; Fujioka, M.; Horiuchi, K.; Satoh, A.O.; Nepal, P.; et al. A Sialylated Voltage-Dependent Ca(2+) Channel Binds Hemagglutinin and Mediates Influenza A Virus Entry into Mammalian Cells. *Cell Host Microbe* **2018**, *23*, 809-818 e805, doi:10.1016/j.chom.2018.04.015.
37. Jitobaom, K.; Tongluan, N.; Smith, D.R. Involvement of voltage-dependent anion channel (VDAC) in dengue infection. *Sci Rep* **2016**, *6*, 35753, doi:10.1038/srep35753.
38. Muller, M.; Slivinski, N.; Todd, E.; Khalid, H.; Li, R.; Karwatka, M.; Merits, A.; Mankouri, J.; Tuplin, A. Chikungunya virus requires cellular chloride channels for efficient genome replication. *PLoS Negl Trop Dis* **2019**, *13*, e0007703, doi:10.1371/journal.pntd.0007703.
39. Hansen, T.H.; Yan, Y.; Ahlberg, G.; Vad, O.B.; Refsgaard, L.; Dos Santos, J.L.; Mutsaers, N.; Svendsen, J.H.; Olesen, M.S.; Bentzen, B.H.; et al. A Novel Loss-of-Function Variant in the Chloride Ion Channel Gene Clcn2 Associates with Atrial Fibrillation. *Sci Rep* **2020**, *10*, 1453, doi:10.1038/s41598-020-58475-9
40. Leisle, L.; Ludwig, C.F.; Wagner, F.A.; Jentsch, T.J.; Stauber, T. ClC-7 is a slowly voltage-gated 2Cl(-)/1H(+)-exchanger and requires Ostm1 for transport activity. *EMBO J* **2011**, *30*, 2140-2152, doi:10.1038/emboj.2011.137.
41. Bourdin, B.; Shakeri, B.; Tetreault, M.P.; Sauve, R.; Lesage, S.; Parent, L. Functional characterization of CaValpha2delta mutations associated with sudden cardiac death. *J Biol Chem* **2015**, *290*, 2854-2869, doi:10.1074/jbc.M114.597930.
42. Yang, L.; Katchman, A.; Morrow, J.P.; Doshi, D.; Marx, S.O. Cardiac L-type calcium channel (Cav1.2) associates with gamma subunits. *FASEB J* **2011**, *25*, 928-936, doi:10.1096/fj.10-172353.
43. Martins-Marques, T.; Catarino, S.; Goncalves, A.; Miranda-Silva, D.; Goncalves, L.; Antunes, P.; Coutinho, G.; Leite Moreira, A.; Falcao Pires, I.; Girao, H. EHD1 Modulates Cx43 Gap Junction Remodeling Associated With Cardiac Diseases. *Circ Res* **2020**, *126*, e97-e113, doi:10.1161/CIRCRESAHA.119.316502.
44. Brink, P.R.; Valiunas, V.; White, T.W. Lens Connexin Channels Show Differential Permeability to Signaling Molecules. *Int J Mol Sci* **2020**, *21*, doi:10.3390/ijms21186943.
45. Ye, W.G.; Yue, B.; Aoyama, H.; Kim, N.K.; Cameron, J.A.; Chen, H.; Bai, D. Junctional delay, frequency, and direction-dependent uncoupling of human heterotypic Cx45/Cx43 gap junction channels. *J Mol Cell Cardiol* **2017**, *111*, 17-26, doi:10.1016/j.yjmcc.2017.07.117.
46. Waldmann, R.; Champigny, G.; Bassilana, F.; Heurteaux, C.; Lazdunski, M. A proton-gated cation channel involved in acid-sensing. *Nature* **1997**, *386*, 173-177, doi:10.1038/386173a0.
47. Pena-Oyarzun, D.; Batista-Gonzalez, A.; Kretschmar, C.; Burgos, P.; Lavandero, S.; Morselli, E.; Criollo, A. New emerging roles of Polycystin-2 in the regulation of autophagy. *Int Rev Cell Mol Biol* **2020**, *354*, 165-186, doi:10.1016/bs.ircmb.2020.02.006.
48. Huttlin, E.L.; Bruckner, R.J.; Paulo, J.A.; Cannon, J.R.; Ting, L.; Baltier, K.; Colby, G.; Gebreab, F.; Gygi, M.P.; Parzen, H.; et al. Architecture of the human interactome defines protein communities and disease networks. *Nature* **2017**, *545*, 505-509, doi:10.1038/nature22366.

49. Brailoiu, E.; Churamani, D.; Cai, X.; Schrlau, M.G.; Brailoiu, G.C.; Gao, X.; Hooper, R.; Boulware, M.J.; Dun, N.J.; Marchant, J.S.; et al. Essential requirement for two-pore channel 1 in NAADP-mediated calcium signaling. *J Cell Biol* **2009**, *186*, 201-209, doi:10.1083/jcb.200904073.
50. Ludwig, A.; Zong, X.; Stieber, J.; Hullin, R.; Hofmann, F.; Biel, M. Two pacemaker channels from human heart with profoundly different activation kinetics. *EMBO J* **1999**, *18*, 2323-2329, doi:10.1093/emboj/18.9.2323.
51. Ahuja, S.; Mukund, S.; Deng, L.; Khakh, K.; Chang, E.; Ho, H.; Shriver, S.; Young, C.; Lin, S.; Johnson, J.P., Jr.; et al. Structural basis of Nav1.7 inhibition by an isoform-selective small-molecule antagonist. *Science* **2015**, *350*, aac5464, doi:10.1126/science.aac5464.
52. Handford, C.A.; Lynch, J.W.; Baker, E.; Webb, G.C.; Ford, J.H.; Sutherland, G.R.; Schofield, P.R. The human glycine receptor beta subunit: primary structure, functional characterisation and chromosomal localisation of the human and murine genes. *Brain Res Mol Brain Res* **1996**, *35*, 211-219, doi:10./0610.0169-328X(95)00218-H [pii].
53. Coverstone, E.D.; Bach, R.G.; Chen, L.; Bierut, L.J.; Li, A.Y.; Lenzini, P.A.; O'Neill, H.C.; Spertus, J.A.; Sucharov, C.C.; Stitzel, J.A.; et al. A novel genetic marker of decreased inflammation and improved survival after acute myocardial infarction. *Basic Res Cardiol* **2018**, *113*, 38, doi:10.1007/s00395-018-0697-7
54. Martina, J.A.; Lelouvier, B.; Puertollano, R. The calcium channel mucolipin-3 is a novel regulator of trafficking along the endosomal pathway. *Traffic* **2009**, *10*, 1143-1156, doi:10.1111/j.1600-0854.2009.00935.x.
